# Photocatalytic Oxygen Evolution from Water Splitting

**DOI:** 10.1002/advs.202002458

**Published:** 2020-11-18

**Authors:** Sen Lin, Hongwei Huang, Tianyi Ma, Yihe Zhang

**Affiliations:** ^1^ Beijing Key Laboratory of Materials Utilization of Nonmetallic Minerals and Solid Wastes National Laboratory of Mineral Materials School of Materials Science and Technology China University of Geosciences Beijing 100083 China; ^2^ Discipline of Chemistry University of Newcastle Callaghan NSW 2308 Australia

**Keywords:** catalytic reaction, charge separation, oxygen evolution, photoabsorption, photocatalysis

## Abstract

Photocatalytic water splitting has attracted a lot of attention in recent years, and O_2_ evolution is the decisive step owing to the complex four‐electrons reaction process. Though many studies have been conducted, it is necessary to systematically summarize and introduce the research on photocatalytic O_2_ evolution, and thus a systematic review is needed. First, the corresponding principles about O_2_ evolution and some urgently encountered issues based on the fundamentals of photocatalytic water splitting are introduced. Then, several types of classical water oxidation photocatalysts, including TiO_2_, BiVO_4_, WO_3_, *α*‐Fe_2_O_3_, and some newly developed ones, such as Sillén–Aurivillius perovskites, porphyrins, metal–organic frameworks, etc., are highlighted in detail, in terms of their crystal structures, synthetic approaches, and morphologies. Third, diverse strategies for O_2_ evolution activity improvement via enhancing photoabsorption and charge separation are presented, including the cocatalysts loading, heterojunction construction, doping and vacancy formation, and other strategies. Finally, the key challenges and future prospects with regard to photocatalytic O_2_ evolution are proposed. The purpose of this review is to provide a timely summary and guideline for the future research works for O_2_ evolution.

## Graphical abstract

Photocatalytic O_2_ evolution reaction is the rate‐determining step in the water splitting process. This review provides a comprehensive summary on the progress of photocatalytic O_2_ evolution from water splitting, involving the fundamentals and mechanism of O_2_ evolution, several typical photocatalysts in terms of their crystal structures, synthetic approaches and morphologies, and diverse strategies for O_2_ evolution activity improvement.

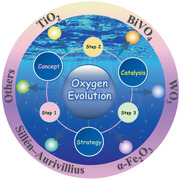

## 1 Introduction

Over the last few decades, the problems of energy shortage and environmental pollution have become the major issues that need to be solved for the sustainable development of modern society and economics.^[^ ^1^ ^]^ Since TiO_2_ photoelectrode was found to be able to decompose H_2_O into H_2_ and O_2_ under UV irradiation in 1972, semiconductor‐based photocatalytic technologies have attracted extensive attentions for environmental purification and energy conversion.^[^ ^2^ ^]^ A large variety of semiconductor photocatalysts have been developed, such as TiO_2_, ZnO, g‐C_3_N_4_, sulfides, bismuth‐based compounds, black phosphorus, etc.,^[^ ^3^, ^4^, ^5^, ^6^, ^7^, ^8^, ^9^ ^]^ which show various applications in the areas of degradation of domestic sewage, industrial dyes and pesticides,^[^ ^10^, ^11^ ^]^ water splitting for H_2_, O_2_, and H_2_O_2_,^[^ ^12^, ^13^, ^14^ ^]^ carbon dioxide conversion for generating CO, CH_4_, HCHO, CH_3_OH, etc.,^[^ ^15^, ^16^ ^]^ nitrogen fixation,^[^ ^17^ ^]^ removal of harmful indoor gases,^[^ ^18^, ^19^ ^]^ solar cell, etc.^[^ ^20^ ^]^ The photocatalytic process generally includes three stages: i) the generation of the electron–hole pairs in the bulk phase of photocatalysts under light irradiation (*hv* ≥ *E*_g_); ii) the migration of electrons and holes from bulk to the surface of photocatalysts, and some of them will be recombined during this process; and iii) the residual electrons and holes on the surface of photocatalysts will react with the adsorbed reactants for reduction and oxidation reactions.^[^ ^21^ ^]^

Photocatalytic overall water splitting (OWS) is a thermodynamically uphill process (Δ*H*_*ϕ*_ = 285.5 KJ mol^−1^), which cannot occur spontaneously. It is composed of two half reactions, namely, the hydrogen evolution reaction (HER) and the oxygen evolution reaction (OER), both of which are the processes of Gibbs free with. It can be realized through the solar light irradiation over photocatalysts, which has become one of the most promising ways to produce clean energy due to the advantages of cleanness, low cost and high physicochemical stability, the equations are as follows^[^ ^22^ ^]^

(1) $$\begin{array}{c} 2H^{+}+2e^{-}\to H_{2}\;\;\left(\mathrm{reduction}\;\mathrm{reaction}\right) \end{array}$$

(2) $$\begin{array}{c} 4OH^{-}+4h^{+}\to O_{2}+2H_{2}O\;\left(\mathrm{oxidation}\;\mathrm{reaction}\right) \end{array}$$

The photocatalytic water splitting reactions are usually carried out in the presence of sacrificial agents to accelerate the reduction/oxidation process in the form of the rapid consumption of photogenerated holes/electrons. The generally used sacrificial agents are methanol, lactic acid, triethanolamine, and Na_2_S–Na_2_SO_3_ for H_2_ evolution, and silver nitrate, ferric chloride, and sodium iodate for O_2_ evolution.^[^ ^6^, ^23^, ^24^, ^25^ ^]^ Compared to H_2_ evolution, the O_2_ evolution is a four‐electrons transferring process with high reaction barrier, which is the rate‐determining step in OWS reaction.^[^ ^22^ ^]^ Considering the extra kinetic overpotential requirement in the actual reaction situation due to the characteristic of multielectron/proton processes of water oxidation, the bandgaps of the used photocatalysts should be generally no smaller than 1.6 eV to conquer the defect of sluggish kinetics, which can be ascribed to the multistep reactions of the existent active intermediates during the O_2_ evolution process. The reaction process is distinct under the acidic or alkaline condition, and the equations are as follows^[^ ^26^ ^]^

(3) $$\begin{array}{c} 2H_{2}O\to O_{2}+4e^{-}+4H^{+}\;\left(\mathrm{acidic}\;\mathrm{condition}\right) \end{array}$$

(4) $$\begin{array}{c} 4OH^{-}\to O_{2}+4e^{-}+2H_{2}O\;\left(\mathrm{alkaline}\;\mathrm{reaction}\right) \end{array}$$

According to the three basic stages of photocatalytic reaction, the efficiency of photocatalytic O_2_ evolution is also mainly determined by light absorption, photogenerated charge separation, and surface catalytic reaction, which can be expressed as follows^[^ ^26^ ^]^

(5) $$\begin{array}{c} \eta \;=\eta_{\mathrm{absorption}}\;\times \;\eta_{\mathrm{separation}}\;\times \;\eta_{\mathrm{reaction}} \end{array}$$

In order to evaluate the photocatalytic activity and physicochemical stability of O_2_ producing photocatalyst, the most common method is to test the turnover frequency (TOF), turnover number (TON), and apparent quantum efficiency (AQE) of the reaction system, and the corresponding formulas are in the following^[^ ^27^, ^28^ ^]^

(6) $$\begin{array}{c} \mathrm{TOF}=\frac{\mathrm{Number}\;\mathrm{of}\;\mathrm{evolved}\;\mathrm{oxygen}\;\mathrm{molecules}}{\mathrm{Number}\;\mathrm{of}\;\mathrm{active}\;\mathrm{sites}\;\times \;\mathrm{reaction}\;\mathrm{time}} \end{array}$$

(7) $$\begin{array}{c} \mathrm{TON}=\frac{\mathrm{Number}\;\mathrm{of}\;\mathrm{evolved}\;\mathrm{oxygen}\;\mathrm{molecules}}{\mathrm{Number}\;\mathrm{of}\;\mathrm{active}\;\mathrm{sites}} \end{array}$$

(8) $$\begin{array}{c} \mathrm{AQE}=\frac{\mathrm{Number}\;\mathrm{of}\;\mathrm{evolved}\;\mathrm{oxygen}\;\mathrm{molecules}}{\mathrm{Number}\;\mathrm{of}\;\mathrm{active}\;\mathrm{sites}}\times 100\% \end{array}$$

In particular, the AQE is the ratio of the moles of generated O_2_ per unit reaction time to the incident photons absorption number at a certain monochromatic wavelength, whereas the conversion efficiencies for O_2_ evolution photocatalysts are distinct based on their different kinds of bandgaps (direct and indirect). Compared with indirect photocatalysts, the direct semiconductor materials have little change in the momentums during the migration process of photogenerated charge carriers after the absorption of incident photons, leading to the faster water oxidation rates than those of indirect ones.^[^ ^26^, ^27^, ^28^ ^]^

**Figure 1:**
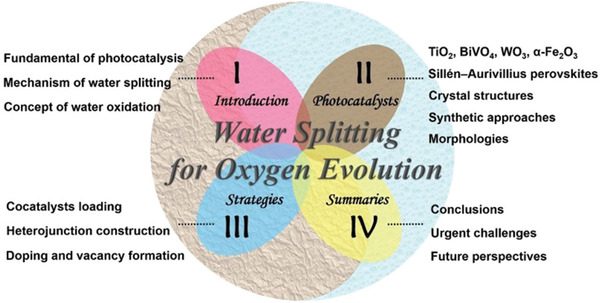
Schematic diagram of general contents in this review.

Previously, the published reviews that are related to the photocatalytic O_2_ evolution from water splitting mainly focused on some specific class of materials, such as BiVO_4_, metal–organic frameworks (MOFs), cobalt complexes, etc.,^[^ ^29^, ^30^, ^31^ ^]^ or limited to the theoretical calculations, defects design from crystal structures, etc.^[^ ^32^, ^33^ ^]^ So far, there is a lack of a systematic overview of the literatures on the photocatalytic O_2_ evolution. Herein, we provide a review for the advances on the researches of photocatalytic O_2_ evolution by starting with the fundamentals of photocatalysis and photocatalytic O_2_ evolution, and then introduce various types of water oxidation photocatalysts based on different crystal structures, compositions, and morphologies. Then, we focus on the diverse strategies that improve the performance of photocatalytic O_2_ evolution. Finally, the challenges and future prospects in this field are proposed. The summary contents are shown in **Figure** **1**.

## 2 Photocatalysts for Oxygen Evolution

**Figure 2:**
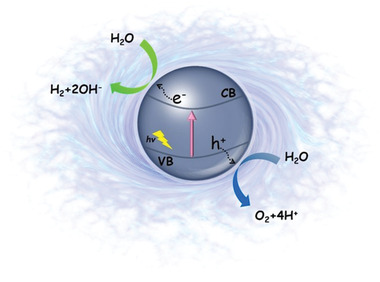
Schematic diagram for photocatalytic water splitting.

As O_2_ evolution reaction is the rate‐determining step during the process of photocatalytic OWS (schematic diagram shown in **Figure** **2**), there are some critical requirements for these high efficiency water oxidation photocatalysts, including: i) the valence band (VB) potentials of the photocatalysts should be higher than those of the O_2_/H_2_O (1.23 eV); ii) to make more use of solar energy, bandgaps of the photocatalysts should be in the range of 1.23–3 eV; iii) the separation of the photogenerated electron–hole pairs within the photocatalysts should be fast under illumination, and then the corresponding redox reactions can occur in time on the surface of photocatalysts.^[^ ^34^, ^35^ ^]^ In addition, the water oxidation active sites should be sufficient on the surface of the photocatalysts in order to restrain the rapid recombination of the photogenerated electrons and holes on the surface. Nowadays, the heterogeneous photocatalysts are still the most commonly researched photocatalysts.

### 2.1 TiO_2_

**Figure 3:**
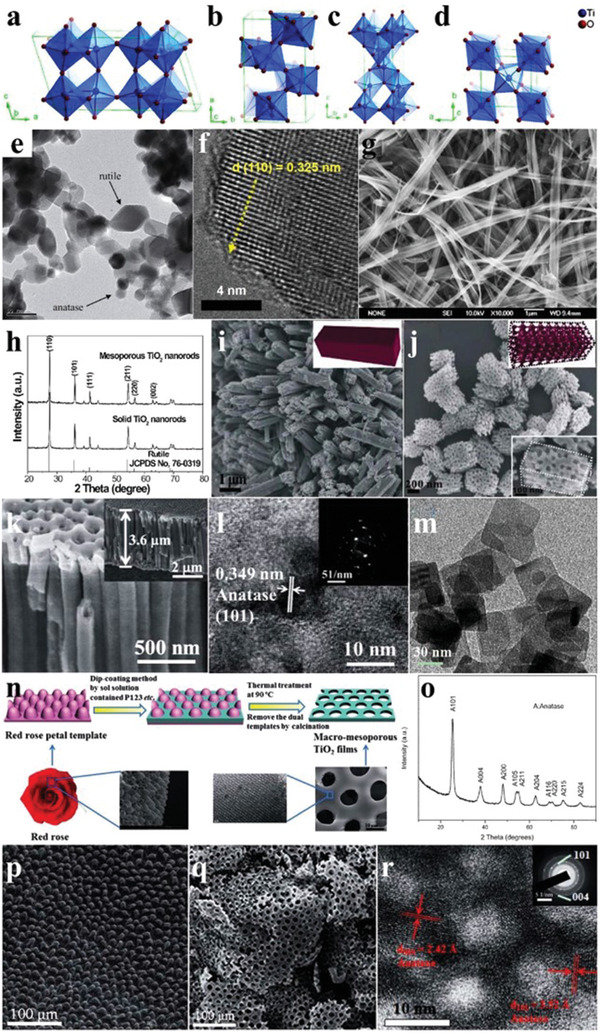
Crystal structures of a) TiO_2_(B), b) brookite, c) anatase, and d) rutile phases of TiO_2_. Reproduced with permission.^[^ ^36^ ^]^ Copyright 2014, American Chemical Society. e) TEM image of P25 nanoparticles. Reproduced with permission.^[^ ^41^ ^]^ Copyright 2015, Elsevier. f) HRTEM image of rutile TiO_2_ nanoparticles. Reproduced with permission.^[^ ^43^ ^]^ Copyright 2015, Springer Nature. g) SEM image of TiO_2_ nanowires. Reproduced with permission.^[^ ^44^ ^]^ Copyright 2008, Elsevier. h) XRD patterns of solid and mesoporous TiO_2_ nanorods. i,j) SEM images of solid and mesoporous TiO_2_ nanorods. Reproduced with permission.^[^ ^45^ ^]^ Copyright 2013, American Chemical Society. k) SEM image, and l) HRTEM image and SAED pattern (inset) of black TiO_2_ nanotubes. Reproduced with permission.^[^ ^46^ ^]^ Copyright 2014, Royal Society of Chemistry. m) TEM image of anatase TiO_2_ nanosheets. Reproduced with permission.^[^ ^47^ ^]^ Copyright 2016, Elsevier. n) Synthesis schematic of mesoporous TiO_2_ flakes. o) XRD pattern of mesoporous TiO_2_ flakes. p,q) SEM images of rose petal and mesoporous TiO_2_ flakes. r) HRTEM image and SAED pattern (inset) of mesoporous TiO_2_ flakes. Reproduced with permission.^[^ ^48^ ^]^ Copyright 2015, Royal Society of Chemistry.

Titanium dioxide (TiO_2_) can be mainly divided into four types: TiO_2_(B), brookite, anatase, and rutile, all of whose crystal structures are composed of TiO_6_ octahedra (**Figure** **3**a–d). As the bandgap of TiO_2_ is 3.2 eV, the photogenerated charge carriers can only be excited by UV light with a wavelength of less than ≈390 nm.^[^ ^36^ ^]^ The most common form of TiO_2_(B) is the layered titanate, and the brookite TiO_2_ belonging to rhombic system has a low physicochemical stability. As anatase TiO_2_ has more oxygen defects inside the crystal and greater band bending to capture the electrons and promote charge separation than the rutile,^[^ ^37^ ^]^ the photocatalytic activity of the anatase TiO_2_ is usually better than that of the rutile phase.^[^ ^38^ ^]^ Commercial TiO_2_ (P25) is composed of both anatase and rutile, which has a higher photocatalytic activity than the monocomponent one, due to the formation of energy barrier at the interface that improves the separation efficiency of the photogenerated charge carriers.^[^ ^39^, ^40^ ^]^

The commonly used routes for synthesis of anatase and rutile TiO_2_ are hydrothermal and calcination methods. In general, the corresponding precursors were first synthesized with some titanium‐containing substances and organic solvents, and then the hydrothermal/solvothermal or calcination processes at different temperatures were utilized to form the anatase, rutile or mixed‐phase TiO_2_,^[^ ^41^, ^42^, ^43^ ^]^ which were confirmed by transmission electron microscopy (TEM) (Figure 3e,f). For instance, with increasing the temperature, the crystalline phase gradually transformed from the TiO_2_(B) to anatase, and finally some of them were transformed into rutile, showing the 1D nanowires morphology with photocatalytic activity higher than that of the P25 nanoparticles due to the large specific surface area (Figure 3g).^[^ ^44^ ^]^ In addition to the conventional preparation methods, researchers have also tried to combine the traditional routes with some additional conditions to synthesize TiO_2_ with novel microstructures. For example, the porous rutile TiO_2_ nanorods were obtained with the synergistic silica as template under the hydrothermal environment, indicating that the crystal growth can be guided under external interference (Figure 3h–j). This 3D porous structure has a larger specific surface area and more exposed active crystal planes than the counterparts with traditional morphology, thus making TiO_2_ more catalytically active.^[^ ^45^ ^]^ 1D black TiO_2_ nanotubes can be obtained through the annealing followed by aluminum reduction treatment, which can produce abundant oxygen vacancies (OVs) on the surface (Figure 3k,l).^[^ ^46^ ^]^ Besides, different dimensions of TiO_2_ can be obtained by the addition of diverse acids under the hydrothermal process, such as the 1D nanorods (by adding hydrochloric acid), 2D nanosheets (by adding hydrofluoric acid) (Figure 3m), etc.^[^ ^47^ ^]^ Similarly, the 2D anatase TiO_2_ flakes with microporous arrays were prepared after preliminary heat treatment and calcination with the rose petals as a natural mesoporous template, rendering TiO_2_ more efficient light absorption and reactive sites, which is known as the petal effect (Figure 3n–r).^[^ ^48^ ^]^

**Figure 4:**
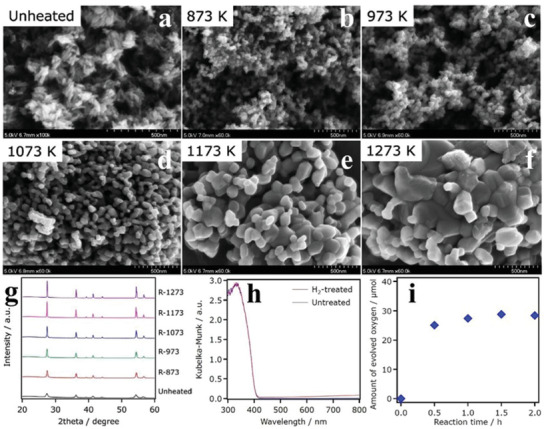
a–f) SEM images of samples calcined at varieties of temperatures. g) XRD patterns of samples calcined at varieties of temperatures. h) DRS of H_2_‐treated/untreated R‐1073. i) Time courses of oxidation evolution for untreated R‐1073. Reproduced with permission.^[^ ^50^ ^]^ Copyright 2014, American Chemical Society.

Compared with anatase TiO_2_, the rutile TiO_2_ has a larger potential in the water oxidation due to the physicochemical characteristics.^[^ ^49^ ^]^ In order to find out the relationship between O_2_ generation capability and physicochemical properties of rutile TiO_2_, a series of TiO_2_ were synthesized by calcination at temperature gradients. It was found that the crystallinity of the samples got better as the calcination temperatures increased (**Figure** **4**g), as confirmed by the scanning electron microscopy (SEM) images that the samples changed from the original amorphous form to the regular grains (Figure 4a–f). The TiO_2_ sample calcined at 1073 K (R‐1073) showed the highest O_2_ evolution rate (44.1 µmol h^−1^ in the FeCl_3_ solution; Figure 4i). The authors speculated that the water oxidation performance of the samples is related to not only the sizes and specific surface areas, but also the OVs. To verify this conjecture, the R‐1073 sample was calcined in the hydrogen (H_2_) atmosphere. There were no significant changes in the crystallinity after treatment, whereas the absorption intensity in the visible region of the H_2_‐treated R‐1073 was slightly higher than before, which may be due to the increase of the OVs concentration in the sample (Figure 4h). Regardless of NaIO_3_ or FeCl_3_ served as the sacrificial agent, the O_2_ evolution performance of R‐1073 after calcination under H_2_ was greatly improved in comparison with the noncalcined one.^[^ ^50^ ^]^

### 2.2 BiVO_4_

In recent years, the n‐type semiconductor bismuth vanadate (BiVO_4_) has attracted wide attention for photocatalytic water oxidation due to the moderate bandgaps (2.3–2.5 eV), suitable VB position, high photochemical stability, and low cost.^[^ ^51^ ^]^

**Figure 5:**
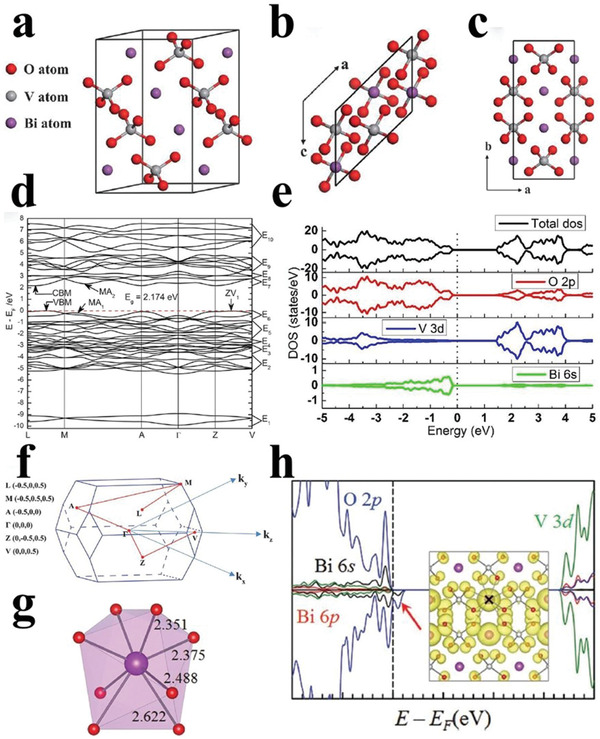
a) The crystal structure, and b) corresponding vertical view and c) side view of BiVO_4_. d) The band structure and f) corresponding brillouin zone of BiVO_4_. Reproduced with permission.^[^ ^52^ ^]^ Copyright 2011, Royal Society of Chemistry. e) The total and projected density of states of BiVO_4_. Reproduced with permission.^[^ ^53^ ^]^ Copyright 2015, Royal Society of Chemistry. g) The bond lengths of Bi—O bonds of BiO_8_ dodecahedra from h) the density of states of nonpolaronic hole states for BiVO_4_. Reproduced with permission.^[^ ^54^ ^]^ Copyright 2013, American Physical Society.

The typical crystalline phase of BiVO_4_ is monoclinic scheelite, of which the crystal structure is composed of the distorted VO_4_ tetrahedron and BiO_8_ dodecahedron, as shown in **Figure** **5**a–c.^[^ ^52^ ^]^ Density functional theory (DFT) calculation results demonstrated that BiVO_4_ is a direct bandgap semiconductor with a bandgap of ≈2.2 eV (Figure 5d,f). It has a higher hole transferring rate than other water oxidation semiconductors, such as In_2_O_3_,^[^ ^52^ ^]^ as verified by the density of states (DOS) (Figure 5e).^[^ ^53^ ^]^ Fascinatingly, the large polaron can be formed under the polaronic state from holes within BiVO_4_, which contributes to the stability of the monoclinic scheelite BiVO_4_ (Figure 5g,h).^[^ ^54^ ^]^

**Figure 6:**
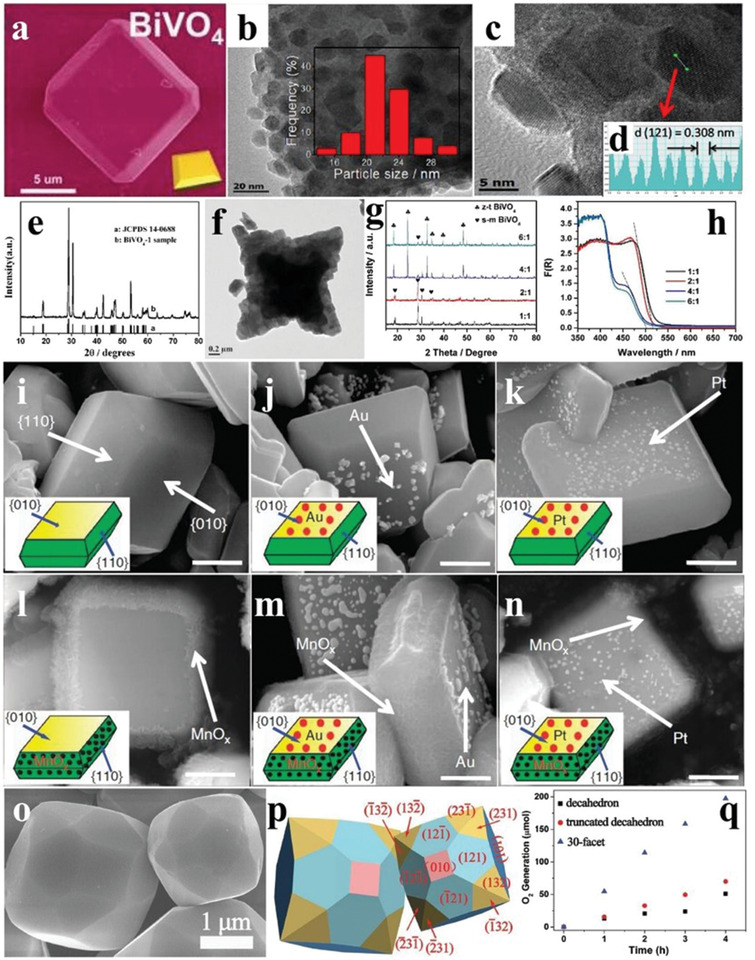
a) SEM image and corresponding diagram (inset) of BiVO_4_. Reproduced with permission.^[^ ^55^ ^]^ Copyright 2018, Royal Society of Chemistry. b) TEM image and size distributions (inset) of BiVO_4_ nanoparticles. c) HRTEM image and d) lattice spacing of BiVO_4_ nanoparticles. Reproduced with permission.^[^ ^56^ ^]^ Copyright 2015, Wiley. e) XRD pattern and f) TEM image of starlike BiVO_4_. Reproduced with permission.^[^ ^57^ ^]^ Copyright 2009, American Chemical Society. g) XRD patterns and h) DRS of BiVO_4_ with Bi/V molar ratios of 1/1, 2/1, 4/1, and 6/1. Reproduced with permission.^[^ ^58^ ^]^ Copyright 2015, Royal Society of Chemistry. i) SEM images of single BiVO_4_ crystals, and BiVO_4_ deposited by j) Au, k) Pt, l) MnO*_x_*, m) Au/MnO*_x_*, and n) Pt/MnO*_x_*; the scale bar is 500 nm. Reproduced with permission.^[^ ^61^ ^]^ Copyright 2013, Springer Nature. o) SEM image and p) corresponding diagram of 30‐faceted BiVO_4_. q) Time courses of oxidation evolution for decahedron, truncated decahedron, and 30‐faceted BiVO_4_. Reproduced with permission.^[^ ^62^ ^]^ Copyright 2017, Wiley.

BiVO_4_ generally has a decahedral shape with smooth surfaces and sharp edges when prepared by hydrothermal reaction with NH_4_VO_3_ and Bi(NO_3_)_3_ as the raw materials, and the diameters of these as‐prepared BiVO_4_ nanoparticles varied considerably depending on the different synthetic conditions (**Figure** **6**a,b).^[^ ^55^, ^56^ ^]^ The lattice spacing of 0.308 nm was attributed to the {121} facets of BiVO_4_ (Figure 6c,d).^[^ ^56^ ^]^ Introduction of a certain amount of chelating agent to the hydrothermal environment usually produced BiVO_4_ with the pure monoclinic phase and a starlike shape (Figure 6e,f).^[^ ^57^ ^]^ Furthermore, the crystalline phases and photoabsorption of BiVO_4_ also varied as the molar ratios of Bi(NO_3_)_3_ and NH_4_VO_3_ changed (Figure 6g,h), leading to modulated photocatalytic properties of BiVO_4_.^[^ ^58^ ^]^

Monoclinic BiVO_4_ shows a large potential for water oxidation.^[^ ^59^, ^60^ ^]^ The active sites of photocatalytic reduction and oxidation reactions of the BiVO_4_ decahedron are closely related to the exposing crystal facets, as revealed by photodeposition of metal/metal oxides.^[^ ^53^ ^]^ As shown in Figure 6i–l, the BiVO_4_ crystals presented a typical regular decahedron structure, and the front and side facets were {010} and {110} facets, respectively. Under illumination, Au and Pt particles were selectively deposited on the {010} facets, while MnO*_x_* was more prone to be deposited on the {110} facets, indicating that the reduction and oxidation active sites were located on the {010} and {110} facets, respectively. The following deposition of Au/MnO*_x_* and Pt/MnO*_x_* was also consistent with the above conclusions (Figure 6m,n).^[^ ^61^ ^]^ As the exposing facets determine the photocatalytic activity, BiVO_4_ with multiple exposing facets was studied. The BiVO_4_ crystals with 30 facets were obtained by a decahedral‐etching engineering under the participation of gold nanoparticles with the controllable concentrations (Figure 6o,p). In addition to the {010} and {110} facets, some uncommon ones, such as {121}, {321}, and {132} facets had also been exposed, offering more possibilities for the photocatalytic activity enhancement of BiVO_4_. The BiVO_4_ with 30 facets showed a higher photocatalytic O_2_ evolution rate, 3–5 times that of decahedron BiVO_4_. The AQE reached 18.3% at 430 nm (Figure 6q).^[^ ^62^ ^]^ Besides exposing facets, the effect of morphology on the photocatalytic activity of BiVO_4_ is another hot topic. By adjusting the pH value of reaction solution, BiVO_4_ with the distinct morphologies were obtained. Under the acidic condition, BiVO_4_ showed a regular decahedron structure, whereas leaf‐like products were obtained in the alkaline media. Owing to the different crystal sizes and bandgaps, these BiVO_4_ crystals synthesized at different pH showed different photocatalytic O_2_ evolution performance, and the BiVO_4_ decahedron exhibited the highest catalytic activity.^[^ ^63^ ^]^

### 2.3 WO_3_

Tungsten trioxide (WO_3_) has been widely used for photo(electro)catalytic water oxidation on account of its suitable bandgap (2.5–2.8 eV), positive VB, and high physicochemical stability.^[^ ^64^, ^65^ ^]^

**Figure 7:**
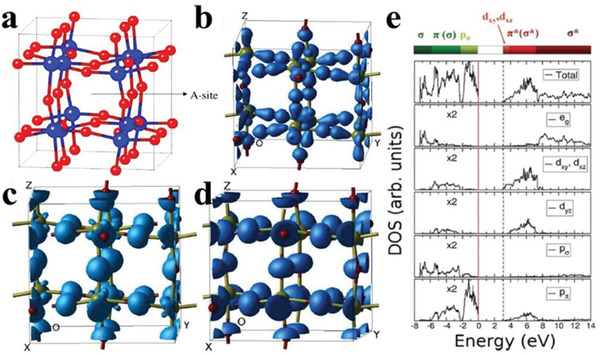
a) The monoclinic structure of WO_3_; blue and red spheres represent W and O atoms, respectively. Reproduced with permission.^[^ ^66^ ^]^ Copyright 2008, American Physical Society. b–d) The electron charge density of WO_3_ from the upper VB. e) The density of states of WO_3_, the zero of energy, and the CB minimum are indicated by the vertical solid line and vertical dashed line, respectively. Reproduced with permission.^[^ ^67^ ^]^ Copyright 2012, American Chemical Society.

The crystal structure of monoclinic WO_3_ is composed of the distorted WO_6_ octahedrons (**Figure** **7**a), in which the marked A site is a good position for doping based on the perovskite‐like structure of WO_3_.^[^ ^66^ ^]^ The top of VB maximum is mainly dominated by the O 2p states (Figure 7e), which can be divided into three parts according to the energy size: low energy region (−7.5 to −6 eV), medium energy region (−6 to −2 eV), and high energy region (−2 to 0 eV) (Figure 7b–d), while the bottom of conduction band (CB) minimum is mainly occupied by the W 5d states (Figure 7e).^[^ ^67^ ^]^ It can also be inferred from the DOS of WO_3_ that doping or defects with metal or nonmetal atoms is one of the effective ways to improve its photocatalytic performance.

**Figure 8:**
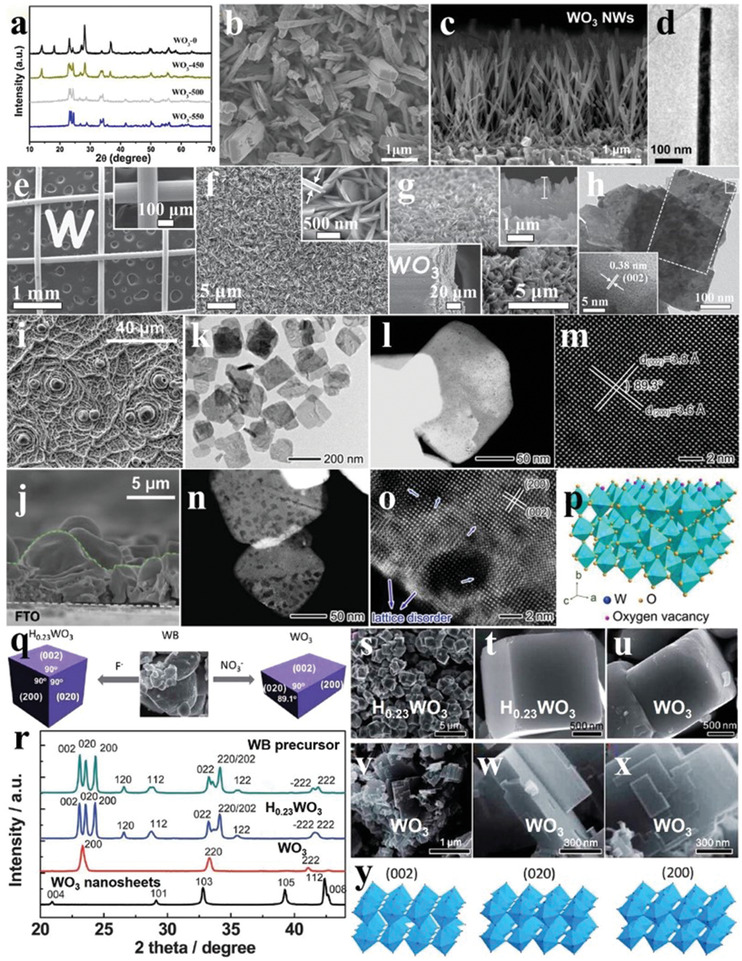
a) XRD patterns of WO_3_ nanorod arrays with heat treatment of different temperatures. b) SEM image of WO_3_ nanorod arrays with calcination treatment of 500 °C. Reproduced with permission.^[^ ^68^ ^]^ Copyright 2016, Elsevier. c) SEM image and d) TEM image of WO_3_ nanowires. Reproduced with permission.^[^ ^69^ ^]^ Copyright 2014, Royal Society of Chemistry. e–g) SEM images of wire mesh and corresponding WO_3_ nanosheet arrays grown on it. h) TEM and HRTEM images (inset) of WO_3_ nanosheet arrays. Reproduced with permission.^[^ ^70^ ^]^ Copyright 2014, Wiley. i,j) SEM images of WO_3_ nanofilms. Reproduced with permission.^[^ ^71^ ^]^ Copyright 2014, Royal Society of Chemistry. k) TEM image, l) STEM image, and m) HAADF‐STEM image of WO_3_ nanosheets with nondefect treatment. n) STEM image and o) HAADF‐STEM image of WO_3_ nanosheets with defect treatment. p) Lattice diagram with OVs of as‐synthesized WO_3_ nanosheets with defect treatment. Reproduced with permission.^[^ ^72^ ^]^ Copyright 2016, American Chemical Society. q) Schematic diagram of the synthesis of H_0.23_WO_3_ crystal and monoclinic WO_3_ nanosheets from tungsten boride precursor. r) XRD patterns of tungsten boride precursor and WO_3_ crystals series. s,t) SEM images of H_0.23_WO_3_ crystals, u) quasi‐cubic‐like WO_3_ crystals, and v–x) monoclinic WO_3_ nanosheets. y) Crystal models of (002), (020), and (200) facets of monoclinic WO_3_. Reproduced with permission.^[^ ^73^ ^]^ Copyright 2012, Royal Society of Chemistry.

Morphology regulation is the mostly involved among the researches of WO_3_ photocatalysts. 1D WO_3_ nanorod arrays on fluorine‐doped tin oxide (FTO) were obtained by the hydrothermal‐calcination method at different temperatures. When the calcination temperature increased to 450 °C, the phase of as‐synthesized 1D WO_3_ nanorod arrays gradually transformed from orthorhombic to monoclinic phase (**Figure** **8**a), as demonstrated by the emergence of (002), (020), and (200) peaks. After calcination at 500 °C, the monoclinic WO_3_ nanorod arrays with the exposed {202} facets were uniformly covered on the FTO substrate (Figure 8b).^[^ ^68^ ^]^ Similar to this convenient method, the 1D WO_3_ nanowire arrays can be also successfully prepared on the FTO substrate under a relatively high temperature (≈550 °C; Figure 8c,d).^[^ ^69^ ^]^ Compared to 1D WO_3_, 3D WO_3_ can promote the separation of photogenerated carriers more effectively. Using the wire mesh as a template, the 3D WO_3_ nanosheets with a thickness of 200 nm were uniformly grown on this scaffold (Figure 8e–g). The exposed {002} facets indicated the successful synthesis of monoclinic phase (Figure 8h).^[^ ^70^ ^]^ In addition to nanosheets, the monoclinic WO_3_ can be deposited on FTO substrate in the form of nanofilms, enriching the 3D WO_3_ materials (Figure 8i,j).^[^ ^71^ ^]^ Due to the structure characteristic of WO_3_ composed of WO_6_ perovskite units, OVs are easier to be introduced into its lattice to obtain the defective 2D nanosheets through calcination in air, which improved the photo(electro)catalytic performance (Figure 8k–p).^[^ ^72^ ^]^

The exposure ratios of the crystal facets have different effects on the photocatalytic O_2_ evolution activity of WO_3_. The monoclinic WO_3_ cubes and WO_3_ nanosheets with different exposing facets were obtained by hydrothermal/calcination treatment of the precursors (Figure 8q). The exposed facets of WO_3_ cubes were mainly {002}, {020}, and {200} facets with exposing ratio of 1:1:1, while that of WO_3_ nanosheets were mainly {002} facets (Figure 8r–y). The normalized O_2_ evolution rate of the WO_3_ cubes reached 5.9 µmol h^−1^, which was more than eight times that of the WO_3_ nanosheets (0.7 µmol h^−1^). In contrast, the WO_3_ nanosheets showed high activity for photoreduction of CO_2_ to CH_4_ (0.34 µmol h^−1^ g^−1^), indicating that the exposing facets have large impacts on the photocatalytic activity of monoclinic WO_3_.^[^ ^73^ ^]^ Besides the facets, the particle size is another factor to influence the photocatalytic activity of WO_3_. For instance, the O_2_ evolution performance of as‐synthesized WO_3_ nanodots, nanoplates, and microcrystals was distinct from each other in the case of similar photoabsorption, among which the WO_3_ nanodots had the highest O_2_ production, implying the direct relationship between the particle sizes and photocatalytic activity of WO_3_.^[^ ^74^ ^]^

### 2.4 *α*‐Fe_2_O_3_

According to the different elemental ratios and crystal structures, iron oxides can be divided into ferrous oxide (FeO), iron trioxide (Fe_2_O_3_), and ferroferric oxide (Fe_3_O_4_), among which the hematite Fe_2_O_3_ (*α*‐Fe_2_O_3_) has a good performance in the photocatalytic field due to its narrow bandgap (1.9–2.2 eV), superior stability, and abundant natural resources compared with other forms of iron oxides.^[^ ^75^, ^76^ ^]^

**Figure 9:**
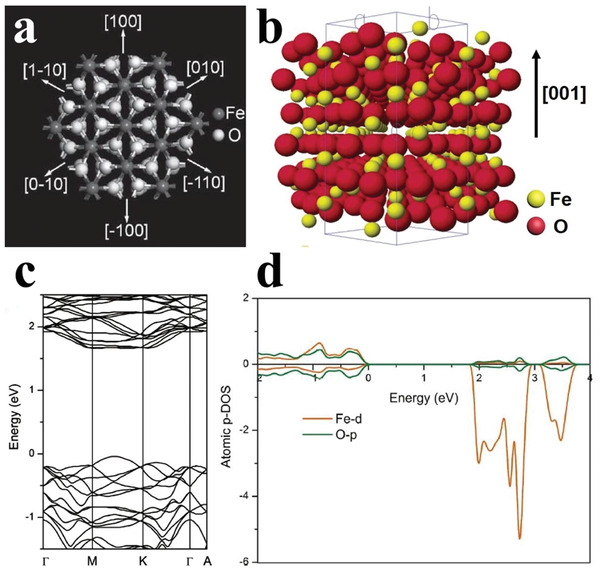
The crystal model of *α*‐Fe_2_O_3_ viewed from a) [001] direction and b) [110] direction. Reproduced with permission.^[^ ^77^, ^78^ ^]^ Copyright 2010, Wiley and 2006, American Chemical Society. c) The band structure and d) partial density of states of *α*‐Fe_2_O_3_. Reproduced with permission.^[^ ^79^ ^]^ Copyright 2010, American Chemical Society.

From the perspective of crystal structure (**Figure** **9**a,b), there are six equivalent crystalline directions in (001) plane in hematite, which are perpendicular to *c*‐axis, based on the Cornell and Schwertmann theory. As the closely packed plane, the crystal growth rates of *α*‐Fe_2_O_3_ along [100] direction and other five equivalent crystalline directions in (001) plane would be more slowly than that of [001] direction, making the crystal of *α*‐Fe_2_O_3_ multifaceted.^[^ ^77^, ^78^ ^]^ As seen from the band structure and DOS of *α*‐Fe_2_O_3_ (Figure 9c,d), the CB minimum is mainly occupied by the Fe 3d orbitals, whereas the O 2p orbitals constitute the VB maximum. According to the electronic structure of *α*‐Fe_2_O_3_, doping with IIIA elements (e.g., Al, Ga, In) in the lattice of *α*‐Fe_2_O_3_ can promote the photocatalytic activity of *α*‐Fe_2_O_3_ and have little effect on its bandgap and band edge energy at the same time, which can be regarded as a good strategy.^[^ ^79^ ^]^

**Figure 10:**
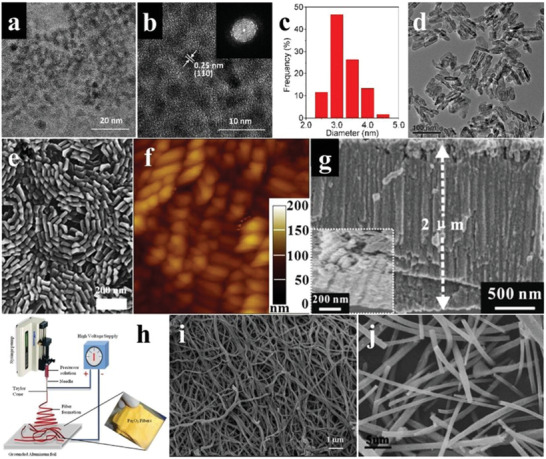
a) TEM image, b) HRTEM image and FFT pattern (inset), and c) size distributions of *α*‐Fe_2_O_3_ quantum dots. Reproduced with permission.^[^ ^80^ ^]^ Copyright 2016, Royal Society of Chemistry. d) TEM image of *α*‐Fe_2_O_3_ nanorods. Reproduced with permission.^[^ ^81^ ^]^ Copyright 2010, American Chemical Society. e) SEM image and f) AFM image of *α*‐Fe_2_O_3_ nanorods arranged on silicon substrates. Reproduced with permission.^[^ ^82^ ^]^ Copyright 2009, Elsevier. g) SEM image and partial enlarged section (inset) of *α*‐Fe_2_O_3_ nanotubes. Reproduced with permission.^[^ ^83^ ^]^ Copyright 2010, Elsevier. h) Schematic diagram of electrostatic spinning. i) SEM image of *α*‐Fe_2_O_3_ nanofibers. Reproduced with permission.^[^ ^84^ ^]^ Copyright 2012, Royal Society of Chemistry. j) SEM image of *α*‐Fe_2_O_3_ nanofibers. Reproduced with permission.^[^ ^85^ ^]^ Copyright 2011, Springer Nature.

**Figure 11:**
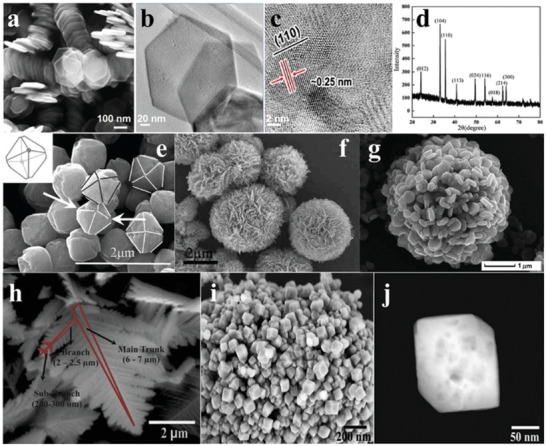
a) SEM image, and b,c) TEM and HRTEM images of *α*‐Fe_2_O_3_ nanosheets. Reproduced with permission.^[^ ^86^ ^]^ Copyright 2017, Wiley. d) XRD pattern and e) SEM image (structural representation inset) of *α*‐Fe_2_O_3_ octahedral nanoparticles. Reproduced with permission.^[^ ^87^ ^]^ Copyright 2012, Royal Society of Chemistry. f) SEM image of *α*‐Fe_2_O_3_ spheres. Reproduced with permission.^[^ ^88^ ^]^ Copyright 2012, Royal Society of Chemistry. g) SEM image of *α*‐Fe_2_O_3_ hollow spheres. Reproduced with permission.^[^ ^89^ ^]^ Copyright 2013, Royal Society of Chemistry. h) SEM image of *α*‐Fe_2_O_3_. i) SEM image and j) dark‐field TEM image of porous *α*‐Fe_2_O_3_ nanocubes. Reproduced with permission.^[^ ^90^ ^]^ Copyright 2013, Springer Nature.

As the O_2_ evolution performance of *α*‐Fe_2_O_3_ can be greatly influenced by the morphology and size, a large number of investigations on the microstructure regulation of *α*‐Fe_2_O_3_ were conducted by using different synthetic techniques under various conditions. The 0D *α*‐Fe_2_O_3_ quantum dots were obtained by the facile microwave‐assisted reverse micelle route (**Figure** **10**a,b). It was shown that the narrow size distribution of as‐synthesized quantum dots was 2–5 nm, which endowed *α*‐Fe_2_O_3_ with higher photocatalytic activity (Figure 10c).^[^ ^80^ ^]^ While 1D *α*‐Fe_2_O_3_ nanorods (Figure 10d–f), nanotubes (Figure 10g) and nanofibers (Figure 10h–j) can be obtained by high‐temperature calcination, deposition, combustion, and electrospinning.^[^ ^81^, ^82^, ^83^, ^84^, ^85^ ^]^ Compared to 0D or 1D *α*‐Fe_2_O_3_, the most widely used strategy for synthesizing 2D and 3D *α*‐Fe_2_O_3_ is the hydrothermal or solvothermal route. 2D hexagonal *α*‐Fe_2_O_3_ nanosheets with a thickness of 15 nm were prepared with a facile solvothermal approach, with the lattice spacing of 0.25 nm for the exposed (110) plane (**Figure** **11**a–c). When they are combined with g‐C_3_N_4_ nanosheets to form a Z‐scheme system, the OWS reaction with H_2_ and O_2_ evolution rate of 38.2 and 19.1 µmol h^−1^ g^−1^, respectively, were achieved.^[^ ^86^ ^]^ The truncated nano‐octahedra structure of nanoparticles were obtained by hydrothermal method (Figure 11d,e), and the average size of these particles was about 800 nm. The uneven edges of these particles increased the exposure of crystal facets and specific surface areas, which were beneficial to the photocatalytic reactions.^[^ ^87^ ^]^ The 3D *α*‐Fe_2_O_3_ are generally constructed by 0D or 2D *α*‐Fe_2_O_3_. For instance, the solid/hollow *α*‐Fe_2_O_3_ nanospheres were always assembled by the nanoparticles or nanosheets through hydrothermal process, leading to the unique hierarchical structure that allowed higher specific surface area and more catalytic active sites compared to nanoparticles or nanosheets (Figure 11f,g).^[^ ^88^, ^89^ ^]^ With potassium ferricyanide as the precursor, *α*‐Fe_2_O_3_ dendrites were synthesized by hydrothermal treatment at 180 °C for 12 h with trunk length of 6–7 µm and branches of 2–2.5 µm (Figure 11h). Under hydrothermal reaction at 180 °C for 12 h, porous *α*‐Fe_2_O_3_ nanocubes with average edge length of 100 nm and various sizes of pores on their surface were fabricated. The hollow structure made *α*‐Fe_2_O_3_ much easier to access reactants for accelerating photocatalytic reactions (Figure 11i,j).^[^ ^90^ ^]^

**Figure 12:**
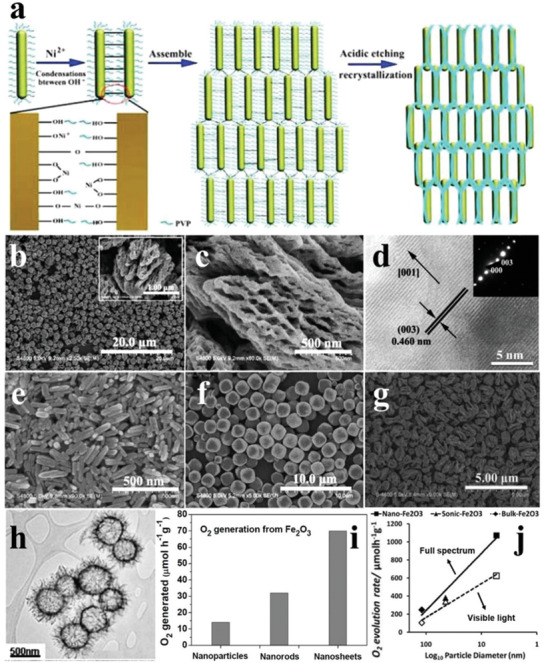
a) Schematic diagram of transition of 1D iron hydroxide nanorods to 3D *α*‐Fe_2_O_3_ hollow nanocages. b,c) SEM images of *α*‐Fe_2_O_3_ hollow nanocages. d) HRTEM image and SAED pattern (inset) of *α*‐Fe_2_O_3_ hollow nanocages. SEM images of hydrothermally synthesized *α*‐Fe_2_O_3_ hollow nanocages with different reaction times of e) 1 h, f) 3 h, and g) 5 h in 200 °C. Reproduced with permission.^[^ ^91^ ^]^ Copyright 2012, Royal Society of Chemistry. h) TEM image of *α*‐Fe_2_O_3_ hollow nanospheres. i) The O_2_ evolution rates of Fe_2_O_3_ nanoparticles, *α*‐Fe_2_O_3_ nanorods, and *α*‐Fe_2_O_3_ hollow nanospheres. Reproduced with permission.^[^ ^92^ ^]^ Copyright 2013, Royal Society of Chemistry. j) Relationship between particle size and oxidation evolution rate for bulk crystals, irregular particles, and single crystalline nanospheres of *α*‐Fe_2_O_3_. Reproduced with permission.^[^ ^93^ ^]^ Copyright 2011, Royal Society of Chemistry.

The transition of dimensions of *α*‐Fe_2_O_3_ can occur during the synthetic process of *α*‐Fe_2_O_3_. As illustrated in **Figure** **12**a, the directional assembly from 1D iron oxide hydroxide chloride nanorods to 3D porous *α*‐Fe_2_O_3_ nanocages was realized by introducing metal ions (Ni^2+^) and surfactant (PVP) as the precursors. The *α*‐Fe_2_O_3_ hollow nanocages were composed of nanorods with rough surfaces. The hematite phase was confirmed by the exposed crystal facets (Figure 12b–d). To study the transformation mechanism during the preparation process, the intermediate products synthesized at different times were observed. As shown in Figure 12e–g, the products gradually changed from nanorods to microspheres and then to hollow nanocages with increasing the reaction time, which may provide a new insight into the morphology regulation of *α*‐Fe_2_O_3_.^[^ ^91^ ^]^ The water oxidation performance of *α*‐Fe_2_O_3_ was also explored in terms of the morphology. The O_2_ evolution rate of the *α*‐Fe_2_O_3_ hollow nanospheres assembled from ultrathin nanosheets reached 70 µmol h^−1^ g^−1^, which was much higher than that of *α*‐Fe_2_O_3_ nanorods (32 µmol h^−1^ g^−1^) and commercial Fe_2_O_3_ nanoparticles (14 µmol h^−1^ g^−1^) (Figure 12h,i), emphasizing the importance of the morphology regulation for the photocatalytic O_2_ evolution performance of *α*‐Fe_2_O_3_.^[^ ^92^ ^]^ similarly, the as‐synthesized single crystalline nanospheres also demonstrated higher O_2_ evolution rate than bulk crystals and irregular particles under either solar or visible light irradiation. Moreover, the O_2_ evolution rates of these catalysts were found to be inversely proportional to the logarithm of the sizes (Figure 12j).^[^ ^93^ ^]^

### 2.5 Sillén–Aurivillius Perovskites

Sillén et al. found a series of compounds that were made up of the [M_2_O_2_]^2+^ metal oxide layers (M = Ca, Sr, Ba, Cd, Li, Na, Sb, Bi) separated by the halide layers and proposed the formula of X*_n_* (*n* = 1, 2, 3) to represent the number of halide layers that separated the metal oxide layers, such as BiPbO_2_X (X = Cl, Br, I) and BiOX (X = Cl, Br, I) are the X_1_ and X_2_ type of structure, respectively. Later, Aurivillius discovered in 1949 that some layered bismuth oxide compounds consist of the alternately stacked [Bi_2_O_2_]^2+^ units and perovskite layers, like Bi_2_MoO_6_, Bi_4_Ti_3_O_12_, Bi_3_TiNbO_9_, etc., the general formula can be described as (Bi_2_O_2_)^2+^(A*_n_*_−1_B*_n_*O_3_ *_n_*_+1_)^2−^, where A and B represent the cations. In recent years, it has been discovered that some compounds possess both of the above two structural characteristics, and they are called Sillén–Aurivillius perovskites; the crystal structures can be expressed as [(Bi_2_O_2_)_2_X]^2+^(A*_n_*_−1_B*_n_*O_3_ *_n_*_+1_)^2−^, where A and B represent the cations and X represents the halide anions. This type of material has recently been found to demonstrate excellent performance of photocatalytic O_2_ evolution from water splitting.^[^ ^94^, ^95^, ^96^, ^97^, ^98^ ^]^

**Figure 13:**
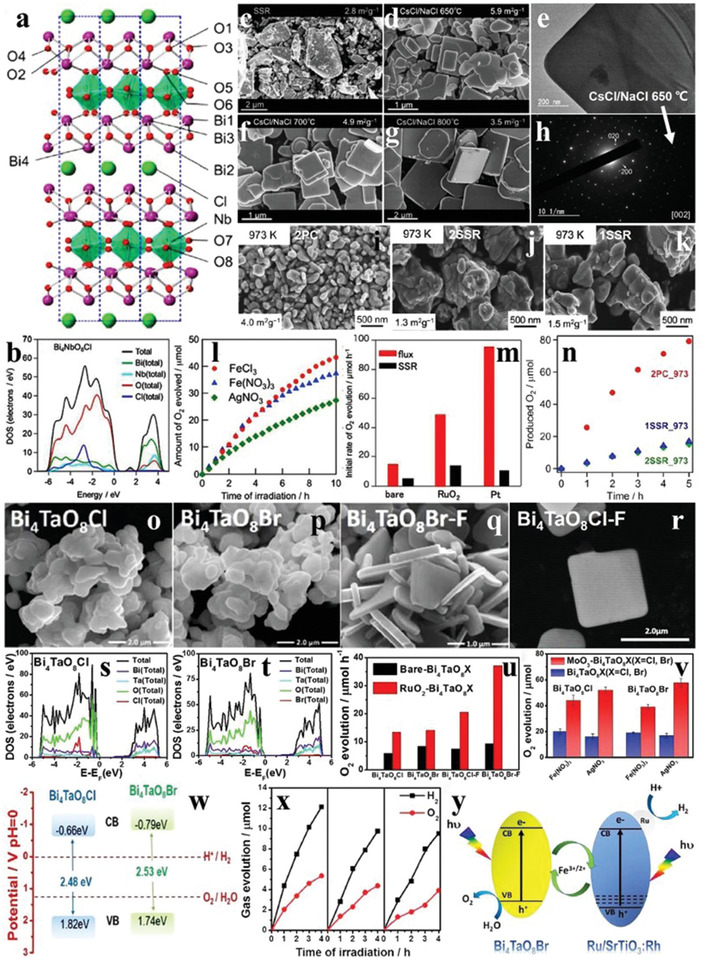
a) Crystal structure of Bi_4_NbO_8_Cl. b) Total and partial density of states of Bi_4_NbO_8_Cl. Reproduced with permission.^[^ ^99^ ^]^ Copyright 2016, American Chemical Society. SEM images of Bi_4_NbO_8_Cl synthesized through c) solid‐state reaction and d,f,g) flux method. e) TEM image and h) SAED pattern of Bi_4_NbO_8_Cl synthesized through flux method. Reproduced with permission.^[^ ^100^ ^]^ Copyright 2019, American Chemical Society. SEM images of Bi_4_NbO_8_Cl synthesized through i) two‐step polymerized complex method, j) solid‐state reaction, and k) conventional single‐step solid‐state reaction. Reproduced with permission.^[^ ^101^ ^]^ Copyright 2018, Royal Society of Chemistry. l) Time courses of oxidation evolution for Bi_4_NbO_8_Cl with different sacrifices. Reproduced with permission.^[^ ^99^ ^]^ Copyright 2016, American Chemical Society. m) The O_2_ evolution rates of pristine Bi_4_NbO_8_Cl, RuO_2_‐loaded Bi_4_NbO_8_Cl, and Pt‐loaded Bi_4_NbO_8_Cl synthesized through flux method and solid‐state reaction. Reproduced with permission.^[^ ^100^ ^]^ Copyright 2019, American Chemical Society. n) Time courses of oxidation evolution for Bi_4_NbO_8_Cl synthesized through two‐step polymerized complex method, solid‐state reaction, and conventional single‐step solid‐state reaction. Reproduced with permission.^[^ ^101^ ^]^ Copyright 2018, Royal Society of Chemistry. SEM images of o) Bi_4_TaO_8_Cl, p) Bi_4_TaO_8_Br, q) flux‐treated Bi_4_TaO_8_Br, and r) flux‐treated Bi_4_TaO_8_Cl. Total and partial density of states of s) Bi_4_TaO_8_Cl and t) Bi_4_TaO_8_Br. u) The O_2_ evolution rates of Bi_4_TaO_8_X and RuO_2_‐loaded Bi_4_TaO_8_X. Reproduced with permission.^[^ ^102^ ^]^ Copyright 2017, Wiley. v) The O_2_ evolution production of Bi_4_TaO_8_X (X = Cl, Br) and MoO_3_–Bi_4_TaO_8_X with Fe(NO_3_)_3_ and Ag(NO_3_)_3_ as the sacrificial agent, respectively. Reproduced with permission.^[^ ^103^ ^]^ Copyright 2018, Wiley. w) Band structures of Bi_4_TaO_8_X (X = Cl, Br). x) Time courses of overall water splitting evolution for Bi_4_TaO_8_Br combined with Ru(0.1 wt%)/Rh‐doped SrTiO_3_ with Fe^3+^/Fe^2+^ as redox cycle mediator. y) Schematic diagram of overall water splitting evolution for Bi_4_TaO_8_Br‐based Z‐scheme system in Fe^3+^/Fe^2+^ redox cycle mediator. Reproduced with permission.^[^ ^102^ ^]^ Copyright 2017, Wiley.

In 2016, Ryu Abe's group first reported the photocatalytic activity for O_2_ production over the Sillén–Aurivillius structured Bi_4_NbO_8_Cl under visible light, which has a layered crystal structure of the alternately stacked [NbO_4_]^6−^ perovskite units and [(Bi_2_O_2_)_2_Cl]^6−^ blocks. Different from some simple oxyhalides, such as BiOCl and BiOBr, the VB maximum of Bi_4_NbO_8_Cl is mainly occupied by the highly dispersive O 2p orbitals and is not prone to self‐oxidation due to the stable oxygen anions, so it has great potential for the catalytic water oxidation (**Figure** **13**a,b).^[^ ^99^ ^]^ In recent years, Abe's group has gained abundant experiences on the synthesis of Sillén–Aurivillius structured photocatalysts. Bi_4_NbO_8_Cl was traditionally synthesized by solid‐state reaction (SSR), which led to the morphology of irregular chunks of the as‐prepared samples (Figure 13c). Based on SSR experience, a certain proportion of alkali metal chlorides (CsCl and NaCl) were be added into the halogen precursors to make up for the halogen volatilization and to provide a solid solution environment (called as flux method). As a result, the Bi_4_NbO_8_Cl nanosheets with well‐defined (00*l*) planes were obtained when calcination temperature reached above the melting points of CsCl and NaCl like 650 °C (Figure 13d,e,h), 700 °C (Figure 13f), or 800 °C (Figure 13g).^[^ ^100^ ^]^ When FeCl_3_ serves as the sacrificial agent, the photocatalytic O_2_ evolution performance of Bi_4_NbO_8_Cl is the best (Figure 13l).^[^ ^99^ ^]^ Importantly, the O_2_ evolution rates of Bi_4_NbO_8_Cl synthesized by the flux method were much higher than those by SSR regardless of with or without the addition of cocatalysts (e.g., RuO_2_, Pt) (Figure 13m).^[^ ^100^ ^]^ Inspiringly, the OWS reaction was realized when Bi_4_NbO_8_Cl was coupled with the H_2_ evolution photocatalyst such as Rh‐doped SrTiO_3_ to construct the Z‐scheme systems, and the system with Bi_4_NbO_8_Cl synthesized by flux method exhibited a higher photocatalytic activity than that prepared by SSR.

The different morphologies of Bi_4_NbO_8_Cl were obtained by the two‐step polymerized complex method (2PC), solid‐state reaction (2SSR), and conventional single‐step solid‐state reaction (1SSR) at the calcination temperature of 973 K (Figure 13i–k), and Bi_4_NbO_8_Cl synthesized by 2PC route had much smaller size and larger specific surface area than the samples synthesized by 2SSR and 1SSR, implying that the morphology of Bi_4_NbO_8_Cl was associated with the synthetic routes. The O_2_ evolution production of Bi_4_NbO_8_Cl prepared by 2PC was also higher than those of samples synthesized by 2SSR and 1SSR routes (Figure 13n). Naturally, the OWS activity of the 2PC synthesized Bi_4_NbO_8_Cl was also higher than that of 1SSR prepared sample when combined with Rh‐doped SrTiO_3_ with the existence of Fe^3+^/Fe^2+^ redox mediator, highlighting the status of O_2_ evolution reaction that as the decisive step for the overall water splitting.^[^ ^101^ ^]^

Besides Bi_4_NbO_8_Cl, other Sillén–Aurivillius perovskites with similar crystal structures were also synthesized, including Bi_4_TaO_8_Cl and Bi_4_TaO_8_Br. The DOS plots in Figure 13s,t stated that the O 2p orbitals were powerfully hybridized with Bi 6s orbitals, which is a common characteristic of the electronic structures of most bismuth‐based materials. Similar to Bi_4_NbO_8_Cl, the morphologies of Bi_4_TaO_8_X (X = Cl, Br) were transformed from the original bulk particles to the regular nanosheets with the thickness of ≈100 nm when the flux method was adopted with NaCl and KCl as the flux agents (Figure 13o–r). The O_2_ evolution rates of Bi_4_TaO_8_X treated with fluxes were higher than that without fluxes. The O_2_ production activity of all the Bi_4_TaO_8_X was promoted after loading the cocatalyst, and flux‐treated Bi_4_TaO_8_X showed larger improvement (Figure 13u).^[^ ^102^ ^]^ The interfacial modulation is considered to be an effective way to enhance the photocatalytic performance of photocatalysts. The O_2_ evolution performances of Bi_4_TaO_8_X were substantially improved after being decorated with MoO_3_ particles by the impregnation route (Figure 13v). After the introduction of MoO_3_, the photogenerated electrons would migrate from the VB of Bi_4_TaO_8_X to the CB of MoO_3_ due to their distinct Fermi levels, thus forming a built‐in electric field to effectively separate the charge carriers. Taking into account that the bandgap positions of both of Bi_4_TaO_8_Cl and Bi_4_TaO_8_Br meet the thermodynamic requirements of photocatalytic water splitting reaction (Figure 13w), when Bi_4_TaO_8_Br was coupled with Rh‐doped SrTiO_3_ to form the Z‐scheme system, the yield ratio of H_2_ to O_2_ was close to 2:1 and it also showed a high stability, which provides an new reference for OWS (Figure 13x,y).^[^ ^103^ ^]^

**Figure 14:**
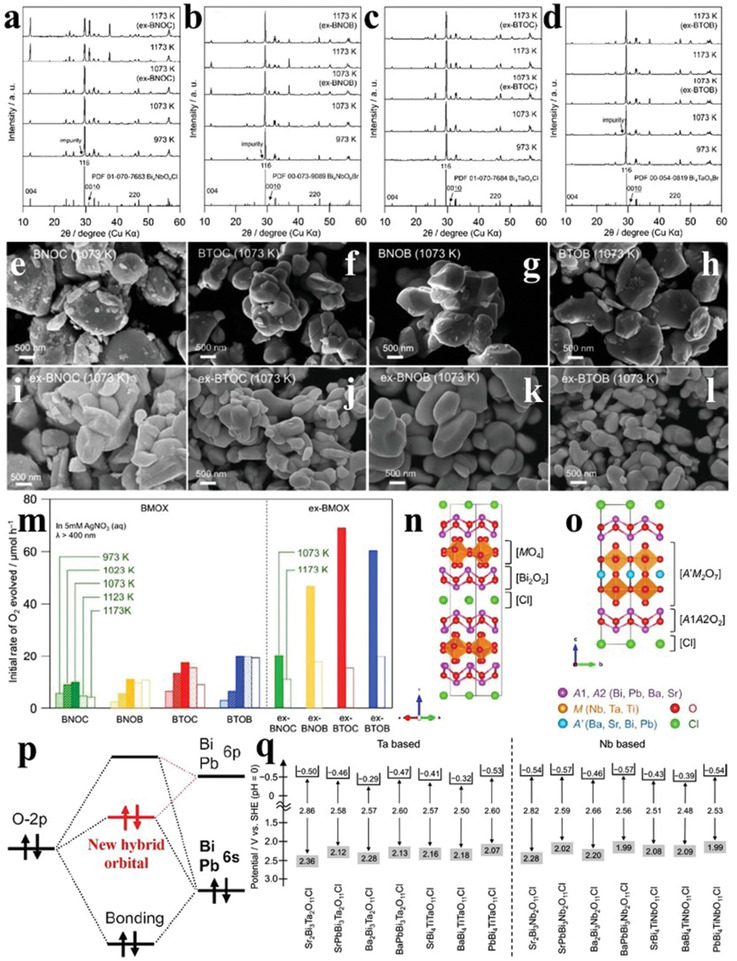
a–d) XRD patterns of Bi_4_MO_8_X and ex‐Bi_4_MO_8_X synthesized with varieties of calcination temperatures. e–l) SEM images of Bi_4_MO_8_X and ex‐Bi_4_MO_8_X calcined at 1073 K. m) The O_2_ evolution rates of Bi_4_MO_8_X and ex‐Bi_4_MO_8_X synthesized with varieties of calcination temperatures. Reproduced with permission.^[^ ^104^ ^]^ Copyright 2018, Royal Society of Chemistry. Crystal models of n) single‐layered perovskite oxyhalides Bi_4_MO_8_Cl and o) double‐layered perovskites A_4_A′M_2_O_11_Cl. p) Schematic diagram of revised‐lone‐pair theory with interaction between O 2p orbitals and Bi/nearby anion/cation 6s and 6p orbitals. q) Bandgaps of double‐layered perovskites A_4_A′M_2_O_11_Cl. Reproduced with permission.^[^ ^105^ ^]^ Copyright 2019, American Chemical Society.

To make up for the volatilization effect of halogen species, excess halogen precursors were added in the SSR process of Bi_4_MO_8_X (M = Nb, Ta; X = Cl, Br) to obtain the samples denoted as ex‐Bi_4_MO_8_X (M = Nb, Ta; X = Cl, Br).^[^ ^100^ ^]^ As can be seen from **Figure** **14**a–d, both Bi_4_MO_8_X and ex‐Bi_4_MO_8_X were pure phase, and the addition of excess halogen precursors did not affect the crystal structure of Bi_4_MO_8_X. When calcination temperature increased to 1073 K, some small‐sized particles appeared in the as‐synthesized Bi_4_MO_8_X, which could be ascribed to the halogen species volatilization. Note that this phenomenon was effectively alleviated for ex‐Bi_4_MO_8_X, indicating that excess halogen species compensated for the volatilization of halogen species (Figure 14e–l). With the increase of calcination temperature, the O_2_ evolution rate of Bi_4_MO_8_X first increased and then decreased, reaching the maximum in 1073 K (Figure 14m), indicating that excessive anionic defects emerged at high temperature was not conducive to the O_2_ evolution activity of Bi_4_MO_8_X. Obviously, the O_2_ evolution rate of ex‐Bi_4_MO_8_X was higher than Bi_4_MO_8_X at each calcination temperature, which was attributed to the less anionic defects in ex‐Bi_4_MO_8_X than in Bi_4_MO_8_X.^[^ ^104^ ^]^

Lately, a new series of double‐layered Sillén–Aurivillius perovskites (denoted as A_4_A′M_2_O_11_Cl, A, A′ = Bi, Pb, Ba, and Sr; M = Ta, Nb, and Ti) were synthesized via the polymerized complex (PC) route by Abe's group (Figure 14n,o). The VB positions of the as‐synthesized novel double‐layered perovskites were generally more negative than those of some metallic oxides due to the interaction between O 2p orbitals and Bi/nearby anion/cation 6s and 6p orbitals (Figure 14p,q). Compared to the single‐layered perovskites Bi_4_MO_8_X, the band levels of A_4_A′M_2_O_11_Cl can be neatly tuned due to the distinct valences of cations in A_4_A′M_2_O_11_Cl (A, A′, M) and their various combinations. The O_2_ evolution activities of A_4_A′M_2_O_11_Cl largely depended on the ratios of Cl/Bi on the surfaces, which were consistent with Bi_4_MO_8_X, and the best O_2_ evolution performance was obtained by Ba_2_Bi_3_Nb_2_O_11_Cl (17.6 µmol h^−1^). Besides, the Z‐scheme system consisting of Ba_2_Bi_3_Nb_2_O_11_Cl and Rh‐doped SrTiO_3_ with Fe^3+^/Fe^2+^ revealed an appreciable OWS AQE of 0.7% at 420 nm, comparable to the oxygen evolution activity of bare Bi_4_TaO_8_Cl.^[^ ^105^ ^]^

### 2.6 Other Photocatalysts for Oxygen Evolution

**Figure 15:**
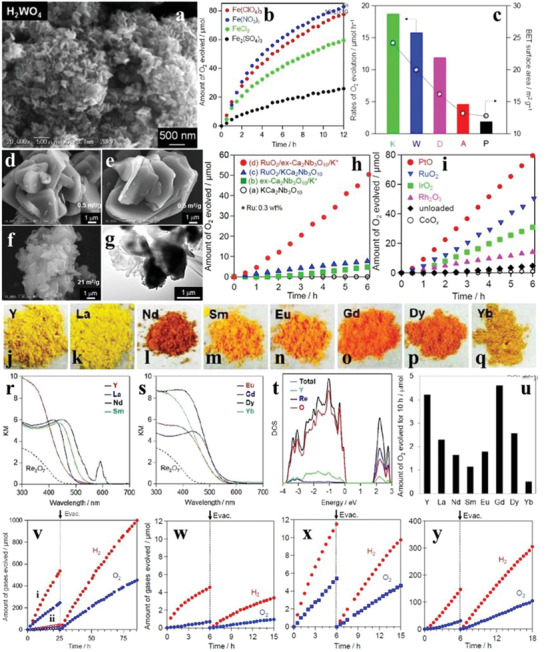
a) SEM image of H_2_WO_4_. b) Time courses of oxidation evolution for H_2_WO_4_ with different sacrifices. c) Specific surface areas and oxidation evolution rates of a series of H_2_WO_4_ samples. Reproduced with permission.^[^ ^106^ ^]^ Copyright 2017, Royal Society of Chemistry. SEM images of d) KCa_2_Nb_3_O_10_ and e) H^+^/KCa_2_Nb_3_O_10_, and f) SEM image and g) TEM image of ex‐Ca_2_Nb_3_O_10_/K^+^. h) Time courses of oxidation evolution for KCa_2_Nb_3_O_10_, ex‐Ca_2_Nb_3_O_10_/K^+^, and RuO_2_‐loaded samples of them. i) Time courses of oxidation evolution for ex‐Ca_2_Nb_3_O_10_/K^+^ loaded with varieties of cocatalysts. Reproduced with permission.^[^ ^107^ ^]^ Copyright 2015, Royal Society of Chemistry. j–q) Digital pictures and r,s) DRS of M_3_ReO_8_. t) Total and partial density of states of Y_3_ReO_8_. u) Amount of oxidation evolution for M_3_ReO_8_ in 10 h. Reproduced with permission.^[^ ^108^ ^]^ Copyright 2017, Royal Society of Chemistry. v) Time courses of overall water splitting evolution for H_2_WO_4_ combined with Ru(0.7 wt%)/Rh‐doped SrTiO_3_ in i) Fe(ClO_4_)_3_ aqueous solution and ii) distilled water. Reproduced with permission.^[^ ^106^ ^]^ Copyright 2017, Royal Society of Chemistry. Time courses of overall water splitting evolution for w) PtO(0.5 wt%)/KCa_2_Nb_3_O_10_ or x) PtO(0.5 wt%)/ex‐Ca_2_Nb_3_O_10_/K^+^ combined with Pt(0.5 wt%)/Rh‐doped SrTiO_3_ in KI aqueous solution. y) Time courses of overall water splitting evolution for ex‐Ca_2_Nb_3_O_10_/K^+^/H^+^ combined with Ru(0.7 wt%)/Rh‐doped SrTiO_3_ in Fe(ClO_4_)_2_ aqueous solution. Reproduced with permission.^[^ ^107^ ^]^ Copyright 2015, Royal Society of Chemistry.

H_2_WO_4_ belongs to the orthorhombic phase and its crystal structure consists of WO_5_(H_2_O) octahedral units, which has a wide absorption region for visible light. It was synthesized by the dehydration of H_4_WO_5_, generating products composed of aggregated plate‐like particles with the sizes of 50–500 nm (**Figure** **15**a). The O_2_ evolution performance of H_2_WO_4_ was the best when using Fe(NO_3_)_3_ as the sacrificial agent, whereas Fe_2_(SO_4_)_3_ was not conducive to the O_2_ evolution production, which was attributed to the fact that it was more easy for Fe^3+^ to form the cation complex with SO_4_^2−^ (Figure 15b). The O_2_ evolution rate of H_2_WO_4_ was directly proportional to its specific surface area, demonstrating that the larger specific surface area favors the reduction of Fe^3+^ (Figure 15c). Similar to the above perovskites, H_2_WO_4_ can also achieve OWS reaction when combined with Rh‐doped SrTiO_3_, and the evolution activity of H_2_ and O_2_ was closely related to the solution environment, e.g., the gas yield in the Fe(ClO_4_)_3_ solution was more higher than that in distilled water (Figure 15v).^[^ ^106^ ^]^

As one kind of cation‐exchangeable layered metal oxides, KCa_2_Nb_3_O_10_ can be easily intercalated by small molecules, which is beneficial for water oxidation. After exfoliating the H^+^/KCa_2_Nb_3_O_10_ (K^+^ of KCa_2_Nb_3_O_10_ was replaced by H^+^) into nanosheets (denoted as ex‐Ca_2_Nb_3_O_10_/K^+^) by continuous stirring, the specific surface area was greatly improved compared to that of KCa_2_Nb_3_O_10_ (Figure 15d–g). With NaIO_3_ as sacrificial agent, the O_2_ evolution performance of the exfoliated samples was higher than that of KCa_2_Nb_3_O_10_ with or without the cocatalyst RuO_2_ (Figure 15h), owing to the more exposed active sites of ex‐Ca_2_Nb_3_O_10_/K^+^. Some other cocatalysts, such as IrO_2_, can also promote the O_2_ evolution of ex‐Ca_2_Nb_3_O_10_/K^+^, and PtO was the most effective one (Figure 15i). The constructed Z‐scheme system of ex‐Ca_2_Nb_3_O_10_/K^+^–SrTiO_3_ exhibited a more efficient OWS reaction with higher H_2_ and O_2_ yields than that of KCa_2_Nb_3_O_10_–SrTiO_3_ in the KI aqueous solution, owing to the lower O_2_ evolution activity and selectivity for KCa_2_Nb_3_O_10_ (Figure 15w,x). Moreover, when RuO_2_ and Fe(ClO_4_)_2_ were selected as the cocatalyst and reaction solution for this Z‐scheme system, respectively, both H_2_ and O_2_ productions were greatly improved (Figure 15x,y).^[^ ^107^ ^]^

The rare‐earth rhenates M_3_ReO_8_ (M = Y, La, Nd, Sm, Eu, Gd, Dy, and Yb) were obtained by the SSR route (Figure 15j–q). Compared to the corresponding oxides Re_2_O_7_, M_3_ReO_8_ had more intense photoabsorption in the visible light region (Figure 15r,s). Taking Y_3_ReO_8_ as the representative, the DOS revealed that the CB minimum of Y_3_ReO_8_ was mainly occupied by Re 5d and O 2p orbitals, while the O 2p orbitals nearly took up the VB maximum of Y_3_ReO_8_ (Figure 15t). Based on the previous findings, the photogenerated carries were easier to be recombined in the band, which was occupied by the R 4f orbitals of RVO_4_ compounds (R = Ce, Pr, Nd, Sm, Eu, Tb, Dy, Ho, Er, Tm, and Yb), thus Gd_3_ReO_8_ and Y_3_ReO_8_ exhibited the higher O_2_ production than other M_3_ReO_8_ samples (Figure 15u).^[^ ^108^ ^]^

**Figure 16:**
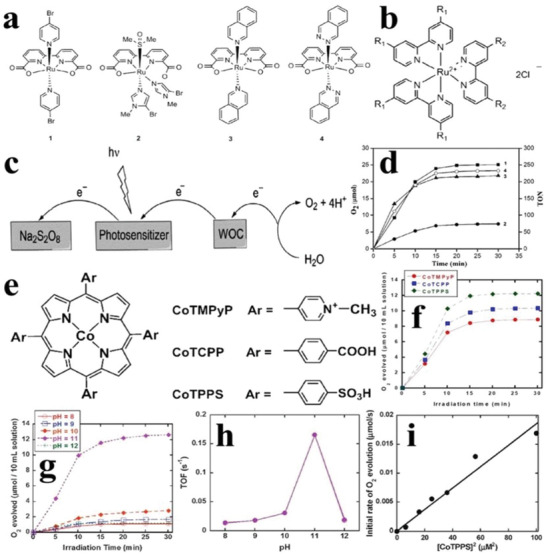
Chemical formulas of a) ruthenium‐based photocatalysts and b) polypyridyl ruthenium photosensitizer [Ru(bpy)_3_]^2+^. c) Schematic diagram of three‐component photocatalytic reaction system. d) Time courses of oxidation evolution and TON for No.1 to No.4 ruthenium‐based photocatalysts from (a). Reproduced with permission.^[^ ^109^ ^]^ Copyright 2013, Elsevier. e) Chemical formulas of cobalt‐based porphyrins photocatalysts. f) Time courses of oxidation evolution for three kinds of cobalt‐based porphyrins from (e). Time courses of g) oxidation evolution and h) TOF for COTPPS in different pH values. i) The O_2_ evolution rates as a function of concentrations of COTPPS. Reproduced with permission.^[^ ^110^ ^]^ Copyright 2013, Royal Society of Chemistry.

In the homogenous system, the onset potentials of some ruthenium‐based compounds (**Figure** **16**a) were lower than those of Ru^3+/2+^ of some normally used photosensitizers, such as polypyridyl ruthenium compounds [Ru(bpy)_3_]^2+^ (R_1_ = COOEt, R_2_ = H) (Figure 16b), so the water oxidation reactions can be driven by [Ru(bpy)_3_]^2+^. In a three‐components photocatalytic reaction system composed of the photocatalyst, photosensitizer, and sacrificial agent (Na_2_S_2_O_8_) (Figure 16c), the No.1 compound in Figure 16a demonstrated the best O_2_ evolution performance (TOF = 20 min^−1^) with the quantum efficiency of 17.1% at 473 nm (Figure 16d),^[^ ^109^ ^]^ and the reaction equations are as follows

(9) $$\begin{array}{c} 4\mathrm{Ru}{\left(\mathrm{bpy}\right)}_{3}^{\;2+}+2S_{2}O_{8}^{\;2-}+2hv\to 4\mathrm{Ru}{\left(\mathrm{bpy}\right)}_{3}^{\;3+}+4{\mathrm{SO}}_{4}^{\;2-} \end{array}$$

(10) $$\begin{array}{c} 4\mathrm{Ru}{\left(\mathrm{bpy}\right)}_{3}^{\;3+}+2H_{2}O\to 4\mathrm{Ru}{\left(\mathrm{bpy}\right)}_{3}^{\;2+}+O_{2}+4H^{+} \end{array}$$

Since some ruthenium‐based compounds have the good water oxidation performance under the assistance of the photosensitizer, some researchers then focused on the specific cobalt‐based porphyrins in the same three‐components reaction system (Figure 16e). The as‐synthesized cobalt(II) tetrakis(*p*‐sulfonatophenyl)porphyrin (COTPPS) showed the highest O_2_ evolution production with the largest TOF of 0.17 s^−1^, when the pH of the reaction solution was 11.0 and [Ru^II^(bpy)_3_](NO_3_)_2_ and Na_2_S_2_O_8_ served as the photosensitizer and sacrificial agent, respectively (Figure 16f–h). Additionally, the O_2_ evolution rate exhibited a linear relationship with the concentration of COTPPS (Figure 16i). The corresponding reaction equations are as follows^[^ ^110^ ^]^

(11) $$\begin{array}{c} Ru^{\mathrm{II}}{\left(\mathrm{bpy}\right)}_{3}^{\;2+}\overset{hv}{\to }Ru^{\mathrm{II}*}{\left(\mathrm{bpy}\right)}_{3}^{\;2+} \end{array}$$

(12) $$\begin{array}{c} Ru^{\mathrm{II}*}{\left(\mathrm{bpy}\right)}_{3}^{\;2+}+S_{2}O_{8}^{\;2-}\to Ru^{\mathrm{III}}{\left(\mathrm{bpy}\right)}_{3}^{\;3+}+{\mathrm{SO}}_{4}^{\;2-}+S{O_{4}}^{-•} \end{array}$$

(13) $$\begin{array}{c} Ru^{\mathrm{II}}{\left(\mathrm{bpy}\right)}_{3}^{\;2+}+{\mathrm{SO}}_{4}^{\;-•}\to Ru^{\mathrm{III}}{\left(\mathrm{bpy}\right)}_{3}^{\;3+}+{\mathrm{SO}}_{4}^{\;2-} \end{array}$$

**Figure 17:**
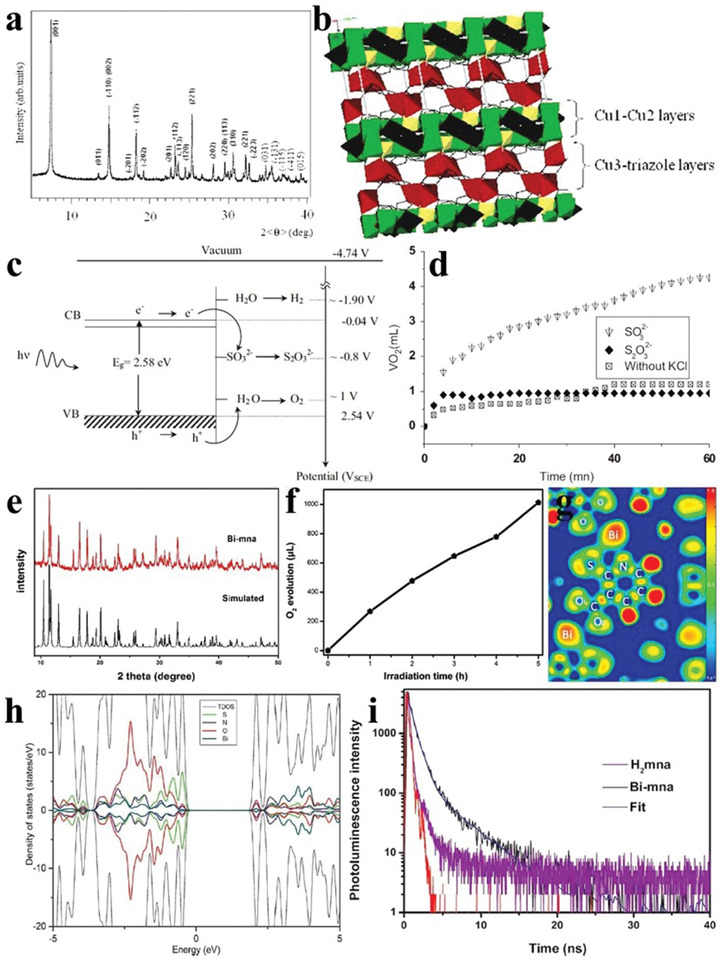
a) XRD pattern of Cu_3_PO_4_(C_2_N_3_H_2_)_2_OH. b) Crystal structure of Cu_3_PO_4_(C_2_N_3_H_2_)_2_OH along [001] direction. c) Schematic diagram of O_2_ evolution of Cu_3_PO_4_(C_2_N_3_H_2_)_2_OH. d) Time courses of oxidation evolution for Cu_3_PO_4_(C_2_N_3_H_2_)_2_OH with/without sacrifices. Reproduced with permission.^[^ ^113^ ^]^ Copyright 2014, Elsevier. e) XRD pattern of Bi‐mna. f) Time courses of oxidation evolution for Bi‐mna. g) Electron localization function plots and h) total and partial density of states of Bi‐mna. i) Time‐resolved fluorescence decay spectra of Bi‐mna and H_2_mna. Reproduced with permission.^[^ ^114^ ^]^ Copyright 2014, Wiley.

As a kind of organic–inorganic hybrid material, MOFs are generally constructed by the self‐assembly of organic ligands and metal ions/clusters through coordination bonds, featuring the advantages of low density, high porosity, and large specific surface area.^[^ ^111^, ^112^ ^]^ In recent years, people have stepped up the researches of water oxidation of MOFs. Cu_3_PO_4_(C_2_N_3_H_2_)_2_OH has a monoclinic crystal structure, in which the copper‐polyhedral layers and copper‐triazole layers were alternately stacked along [001] direction to form a 3D structure (**Figure** **17**a,b). The more positive VB potential than O_2_/H_2_O endowed Cu_3_PO_4_(C_2_N_3_H_2_)_2_OH with the potential of superior water oxidation (Figure 17c). As seen from Figure 17d, the O_2_ evolution performance of Cu_3_PO_4_(C_2_N_3_H_2_)_2_OH reached ≈4.2 mL after 1 h photoirradiation with the addition of sulfite as sacrificial agent.^[^ ^113^ ^]^

As introduced above, the bismuth‐based photocatalysts have become a series of potential materials for water oxidation due to the abundant resources and environment‐friendly feature. Bi^3+^ ions easily coordinate with the organic ligands, so the Bi‐based MOFs are ideal candidates worth to be explored. For instance, a orthorhombic bismuth‐based metal–organic framework (denoted as Bi‐mna) was obtained by solvothermal method with Bi(NO_3_)_3_·5H_2_O and 2‐mercaptonicotinic acid as the precursors (Figure 17e). The O_2_ evolution rate reached 216 µL h^−1^ with AgNO_3_ as the sacrificial agent (Figure 17f). The calculated result of the electron localization function in Figure 17g indicated that the Bi atoms allowed the increase of delocalization of electrons in the covalent bond, which facilitates the migration of charge carriers. The Fukui functions showed that the photogenerated electrons were transferred from S atoms to pyridine rings with Bi atoms as the bridge, and this conclusion was also confirmed by the DOS of Bi‐mna (Figure 17h). Furthermore, the measured time‐resolved fluorescence decay spectra revealed that the average lifetime of Bi‐mna (1.1 ns) was much longer than H_2_mna (100 ps), indicating that Bi‐mna was able to more efficiently separate photogenerated charge carriers (Figure 17i).^[^ ^114^ ^]^ The O_2_ evolution production performance of non‐strategic photocatalysts under distinct reaction conditions is shown in **Table** **1**.

**Table 1:** The O_2_ evolution production performance of photocatalysts under different conditions

Photocatalyst	Sacrificial agent	Amount of sacrificial agent	Light source	Incident light [nm]	Oxygen production rate	AQE	Refs.
TiO_2_	FeCl_3_	10 × 10^−3^ m	300 W Xe	≥350	44.1 µmol h^−1^	–	^[^ ^50^ ^]^
BiVO_4_	NaIO_3_	20 × 10^−3^ m	300 W Xe	≥420	57 µmol h^−1^	18.3% at 430 nm	^[^ ^62^ ^]^
BiVO_4_	AgNO_3_	50 × 10^−3^ m	Sunlight	–	82.8 µmol h^−1^ g^−1^	–	^[^ ^63^ ^]^
BiVO_4_	AgNO_3_	50 × 10^−3^ m	300 W Xe	≥420	310 µmol h^−1^	–	^[^ ^115^ ^]^
BiVO_4_	H_3_PMo_12_O_40_	10 × 10^−3^ m	300 W Xe	≥400	1.03 µmol h^−1^	–	^[^ ^116^ ^]^
BiVO_4_	K_2_S_2_O_8_	11 mg	Sunlight	–	≈4.32 µmol h^−1^	–	^[^ ^117^ ^]^
BiVO_4_	AgNO_3_	50 × 10^−3^ m	300 W Xe	≥420	≈60 µmol h^−1^	–	^[^ ^118^ ^]^
BiVO_4_	AgNO_3_	10 × 10^−3^ m	300 W Xe	≥400	533 µmol h^−1^ g^−1^	–	^[^ ^119^ ^]^
WO_3_	AgNO_3_	850 mg	300 W Xe	≥400	5.9 µmol h^−1^	–	^[^ ^73^ ^]^
WO_3_	NaIO_4_	10 × 10^−3^ m	300 W Xe	≥400	31.6 µmol h^−1^	–	^[^ ^74^ ^]^
WO_3_	FeCl_3_	8 × 10^−3^ m	300 W Xe	≥400	≈10 µmol h^−1^	2.8% at 420 nm	^[^ ^120^ ^]^
WO_3_	KIO_3_	2.5 × 10^−3^ m	300 W Tu	≥420	1256.3 µmol h^−1^	–	^[^ ^121^ ^]^
Fe_2_O_3_	Na_2_S_2_O_8_	60 mg	300 W Xe	≥420	70 µmol h^−1^ g^−1^	–	^[^ ^92^ ^]^
Fe_2_O_3_	AgNO_3_	20 × 10^−3^ m	300 W Xe	≥400	1071 µmol h^−1^ g^−1^	0.61% at 375 nm	^[^ ^93^ ^]^
Bi_4_NbO_8_Cl	FeCl_3_	5 × 10^−3^ m	300 W Xe	≥400	≈4.2 µmol h^−1^	0.4% at 420 nm	^[^ ^99^ ^]^
Bi_4_NbO_8_Cl	FeCl_3_	5 × 10^−3^ m	300 W Xe	≥400	≈2.55 µmol h^−1^	–	^[^ ^122^ ^]^
Bi_4_TaO_8_Cl	FeCl_3_	5 × 10^−3^ m	300 W Xe	≥400	≈16 µmol h^−1^	0.9% at 420 nm	^[^ ^101^ ^]^
Bi_4_TaO_8_Br	AgNO_3_	5 × 10^−3^ m	300 W Xe	≥420	≈37.5 µmol h^−1^	22.3% at 420 nm	^[^ ^102^ ^]^
Bi_4_TaO_8_Cl	AgNO_3_	5 × 10^−3^ m	300 W Xe	≥400	≈68 µmol h^−1^	2.3% at 420 nm	^[^ ^104^ ^]^
Ba_2_Bi_3_Nb_2_O_11_Cl	FeCl_3_	5 × 10^−3^ m	300 W Xe	≥400	17.6 µmol h^−1^	0.7% at 420 nm	^[^ ^105^ ^]^
PbBiO_2_Cl	Fe(NO_3_)_3_	5 × 10^−3^ m	300 W Xe	≥400	≈2.28 µmol h^−1^	0.9% at 400 nm	^[^ ^123^ ^]^
Bi_6_NbWO_14_Cl	AgNO_3_	5 × 10^−3^ m	300 W Xe	≥400	≈5.08 µmol h^−1^	1.4% at 420 nm	^[^ ^124^ ^]^
H_2_WO_4_	Fe(NO_3_)_3_	5 × 10^−3^ m	300 W Xe	≥400	≈6.83 µmol h^−1^	–	^[^ ^106^ ^]^
Gd_3_ReO_8_	AgNO_3_	10 × 10^−3^ m	300 W Xe	≥400	≈0.45 µmol h^−1^	–	^[^ ^108^ ^]^
[Ru(bda)L_2_]^a)^	Na_2_S_2_O_8_	10 × 10^−3^ m	300 W Xe	≥400	≈50 µmol h^−1^	17.1% at 473 nm	^[^ ^109^ ^]^
COTPPS	Na_2_S_2_O_8_	5 × 10^−3^ m	300 W Xe	≥400	≈24.4 µmol h^−1^	–	^[^ ^110^ ^]^
Cu_3_PO_4_(C_2_N_3_H_2_)_2_OH	S_2_O_3_^2−^	0.01 × 10^−3^ m	200 W Tu	≥400	4 mL h^−1^	0.13% at 420 nm	^[^ ^113^ ^]^
Bi‐mna^b)^	AgNO_3_	100 mg	300 W Xe	≥420	216 µL h^−1^	–	^[^ ^114^ ^]^
CoFPS	Na_2_S_2_O_8_	1 × 10^−3^ m	300 W Xe	≥400	0.05 µmol s^−1^	–	^[^ ^125^ ^]^

^a)^ H2bda = 2,2′‐bipyridine‐6,6′‐dicarboxylic acid; L = *N*‐cyclic aromatic ligands

^b)^ mna = 2‐mercaptonicotinic acid.

## 3 Strategies for Enhancing Photocatalytic Oxygen Evolution

The challenges of photocatalytic O_2_ evolution with the participation of sacrificial agents are mainly from the inferior kinetics of the four‐electron migration of water oxidation and intrinsic drawbacks of photocatalysts, such as the low utilization of visible light, easy recombination of photogenerated carriers, and self‐oxidation poisoning from photogenerated holes.^[^ ^126^, ^127^ ^]^ To overcome the disadvantages originating from the above processes, the researchers have put forward various strategies, including cocatalysts loading, heterojunction fabrication, doping and defects formation, and others strategies, such as formation of special microstructure, surface modification, and solid solution construction.

### 3.1 Cocatalysts Loading

In the photocatalytic water splitting process, oxidation reaction requires more rigorous thermodynamic and kinetic conditions. As a small amount of cocatalysts can provide the active sites and trap the photogenerated charge carriers, they can reduce the activation energy and enhance the photocatalytic performance.^[^ ^128^ ^]^ Therefore, the strategy of loading suitable cocatalyst on the surface of semiconductor is necessary. For the O_2_ evolution reaction, the cocatalysts can be divided into the noble metals (e.g., Ag, Ru, Rh, Ir), noble metal oxides (e.g., RuO_2_, RhO_2_, IrO_2_), and transition metal oxides (TMOs) (e.g., MnO_2_, Co_3_O_4_).^[^ ^129^, ^130^ ^]^

**Figure 18:**
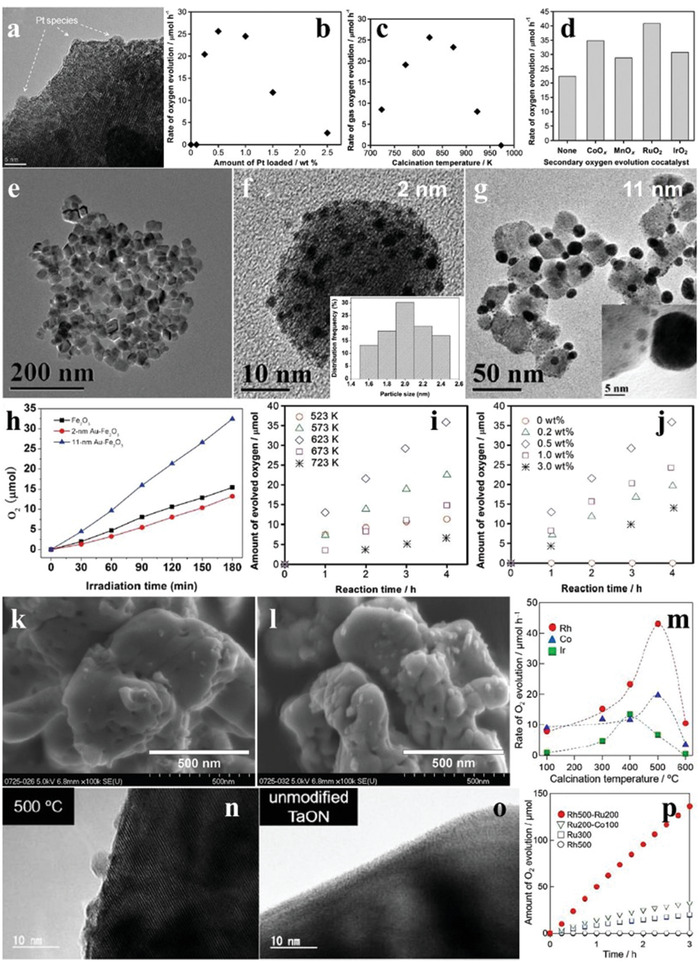
a) TEM image of WO_3_ loaded with 0.5 wt% Pt. b) Time courses of oxidation evolution for WO_3_ as a function of loading amount of Pt. c) Time courses of oxidation evolution for WO_3_ as a function of calcination temperatures. d) The O_2_ evolution rates of pristine Pt–WO_3_ and Pt–WO_3_ loaded with various kinds of second oxidative cocatalysts. Reproduced with permission.^[^ ^131^ ^]^ Copyright 2012, Royal Society of Chemistry. e) TEM image of pristine *α*‐Fe_2_O_3_ nanoparticles. TEM images of f) 2 nm (size distributions inset) and g) 11 nm Au‐loaded *α*‐Fe_2_O_3_. h) Time courses of oxidation evolution for pristine *α*‐Fe_2_O_3_ and Au‐loaded *α*‐Fe_2_O_3_. Reproduced with permission.^[^ ^132^ ^]^ Copyright 2012, Royal Society of Chemistry. i) Time courses of oxidation evolution for TaON loaded with 0.5 wt% Ru at varieties of calcination temperatures. j) Time courses of oxidation evolution for TaON with various loading amounts of Ru. SEM images of TaON calcined at k) 623 K and l) 723 K, respectively. Reproduced with permission.^[^ ^135^ ^]^ Copyright 2011, American Chemical Society. m) The O_2_ evolution rates of TaON loaded with Rh, Co, and Ir as a function of calcination temperatures. TEM images of TaON n) loaded with 1 wt% Rh and o) pure sample. p) Time courses of oxidation evolution for TaON loaded/coloaded with Rh, Ru, and Co at varieties of calcination temperatures. Reproduced with permission.^[^ ^136^ ^]^ Copyright 2019, Royal Society of Chemistry.

Pt is the mostly used noble metal to decrease the overpotential and to promote the charge separation by collecting electrons. For example, when the loading amount of Pt was 0.5 wt% at 823 K by the impregnation method, the uniformly dispersed Pt species were observed clearly on the surface of WO_3_ (**Figure** **18**a), and the as‐synthesized PtO*_x_*/WO_3_ exhibited the highest O_2_ evolution activity (Figure 18b,c). The secondary loading of metal oxides (CoO*_x_*, MnO*_x_*, RuO_2_, IrO_2_) further boosted the O_2_ evolution performance of PtO*_x_*/WO_3_, and the most effective one was RuO_2_ with a O_2_ evolution rate of 41 µmol h^−1^ (Figure 18d).^[^ ^131^ ^]^ Au nanoparticles with diameters of ≈2 and ≈11 nm were in situ loaded on the surface of *α*‐Fe_2_O_3_ particles by photodeposition (Figure 18e–g). The *α*‐Fe_2_O_3_ loaded with larger size Au species showed higher O_2_ evolution production, due to the stronger surface plasmon resonance (SPR) effect (Figure 18h).^[^ ^132^ ^]^ As a typical nitrogen oxide, TaON has attracted widespread attention in water oxidation by virtue of the suitable band position and intense visible light absorption.^[^ ^133^, ^134^ ^]^ To explore the influence of cocatalyst on the O_2_ evolution performance of TaON, Ru‐loaded TaON was synthesized by the impregnation method. It was found that the O_2_ evolution production of TaON reached the maximum when the calcination temperature and the loading amount of Ru was 623 K and 0.5 wt%, respectively (Figure 18i,j). SEM images revealed that the Ru precursor was mostly transformed into RuO_2_ with grain size of ≈30 nm when the calcination temperature was 623 K (Figure 18k), while the agglomeration of RuO_2_ appeared upon higher calcination temperature (Figure 18l), which led to the decreased O_2_ evolution activity of TaON. When Pt/ZrO_2_/TaON and RuO_2_/TaON were separately used as the reduction and oxidation catalysts to form a Z‐scheme system, the yield of H_2_ and O_2_ was close to 2:1, achieving the OWS in NaI aqueous solution.^[^ ^135^ ^]^ On the basis of aforementioned research work, the O_2_ evolution activity of TaON loaded with other metal cocatalysts (Rh, Co, Ir) by the impregnation route was also explored. As shown in Figure 18m–o, the most effective cocatalyst for the O_2_ evolution of TaON was Rh species, and the rate reached ≈43 µmol h^−1^ when the calcination temperature was 500 °C. In addition, the O_2_ evolution rate of TaON loaded with two cocatalysts was higher than those of solely loaded samples, demonstrating the superiority of dual‐loading of cocatalysts (Figure 18p). Besides, the Z‐scheme system consisting of Rh/Ru/TaON and Pt/ZrO_2_/TaON showed higher photocatalytic efficiency than that of solely loaded TaON.^[^ ^136^ ^]^

**Figure 19:**
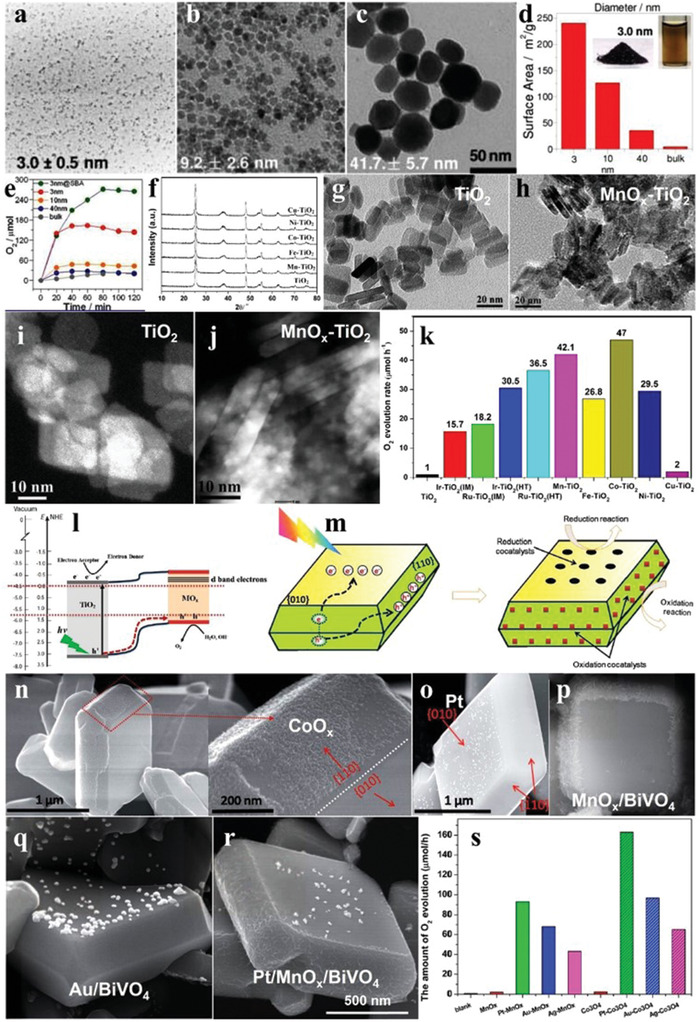
a–c) TEM images and d) surface areas of Co_3_O_4_ nanoparticles (digital pictures of Co_3_O_4_ nanoparticles with diameter of 3 nm in inset). e) Time courses of oxidation evolution for a series of Co_3_O_4_ samples. Reproduced with permission.^[^ ^137^ ^]^ Copyright 2013, American Chemical Society. f) XRD patterns and g,h) SEM images of pristine TiO_2_ nanosheets and MnO*_x_*‐loaded TiO_2_ nanosheets. STEM images of i) pristine TiO_2_ nanosheets and j) MnO*_x_*‐loaded TiO_2_ nanosheets. k) The O_2_ evolution rates and l) schematic diagram of reaction mechanism for pristine TiO_2_ nanosheets and various kinds of cocatalyst‐loaded TiO_2_ nanosheets. Reproduced with permission.^[^ ^140^ ^]^ Copyright 2013, American Chemical Society. m) Schematic diagram of crystal facets selection of reduction/oxidation cocatalysts by photodeposition. SEM images of BiVO_4_ loaded with n) CoO*_x_*, o) Pt, p) MnO*_x_*, q) Au, and r) Pt/MnO*_x_* on {110}/{010} facets of BiVO_4_. s) The O_2_ evolution rates of MnO*_x_*/Co_3_O_4_‐loaded BiVO_4_ deposited by varieties of reduction cocatalysts. Reproduced with permission.^[^ ^142^ ^]^ Copyright 2014, Royal Society of Chemistry.

Considering the rare resources and high cost of noble metals, some common transition metals have been employed as cocatalysts to replace the noble metals in recent years. As shown in **Figure** **19**a–c, Co_3_O_4_ nanoparticles with diameters of 3, 10, and 40 nm were fabricated, in which the 3 nm Co_3_O_4_ presented a good colloidal stability in solution (Figure 19d). Though high O_2_ evolution activity was obtained, it decreased for all the Co_3_O_4_ after a period of time due to the flocculation effect. Fortunately, this phenomena can be alleviated by using porous SBA‐15 as the a carrier (Figure 19e).^[^ ^137^ ^]^ The loading of TMOs is a good strategy to improve the photocatalytic activity of TiO_2_.^[^ ^138^, ^139^ ^]^ When TMOs were loaded on the surface of the TiO_2_ nanosheets through solvothermal method (Figure 19f–h). It can be seen from the scanning transmission electron microscopy (STEM) images that there were irregular clusters (MnO*_x_*) on the surface of the TiO_2_ nanosheets compared to pristine TiO_2_ nanosheets (Figure 19i,j). The very similar phenomenon was also observed for other cocatalysts. Interestingly, the O_2_ evolution rate of TiO_2_ nanosheets loaded with CoO*_x_* (47 µmol h^−1^) was higher than that of Ru/Ir‐loaded counterparts, reflecting the superiority of TMO cocatalysts (Figure 19k,l).^[^ ^140^ ^]^

Since the photogenerated electrons and holes have the feature of selective migration based on different crystal facets of some specific semiconductors (e.g., BiVO_4_),^[^ ^141^ ^]^ it is significant to explore the distributions of different types of cocatalysts on the crystal facets. Under photoirradiation, the Pt and CoO*_x_* were respectively accumulated on {010} and {110} facets of BiVO_4_ (Figure 19m–o), which was distinct from the random deposition of cocatalysts resulted by the impregnation method. The selective photodeposition feature of BiVO_4_ was also confirmed by other single deposition (MnOx, Au) (Figure 19p,q) and dual deposition (Pt and MnO*_x_*) (Figure 19r). Notably, the O_2_ evolution performance of BiVO_4_ loaded with MnO*_x_* or Co_3_O_4_ was improved by deposition of Pt, Au, and Ag (Figure 19s), attributing to the synergetic effect of the reductive and oxidative cocatalysts.^[^ ^142^ ^]^

**Figure 20:**
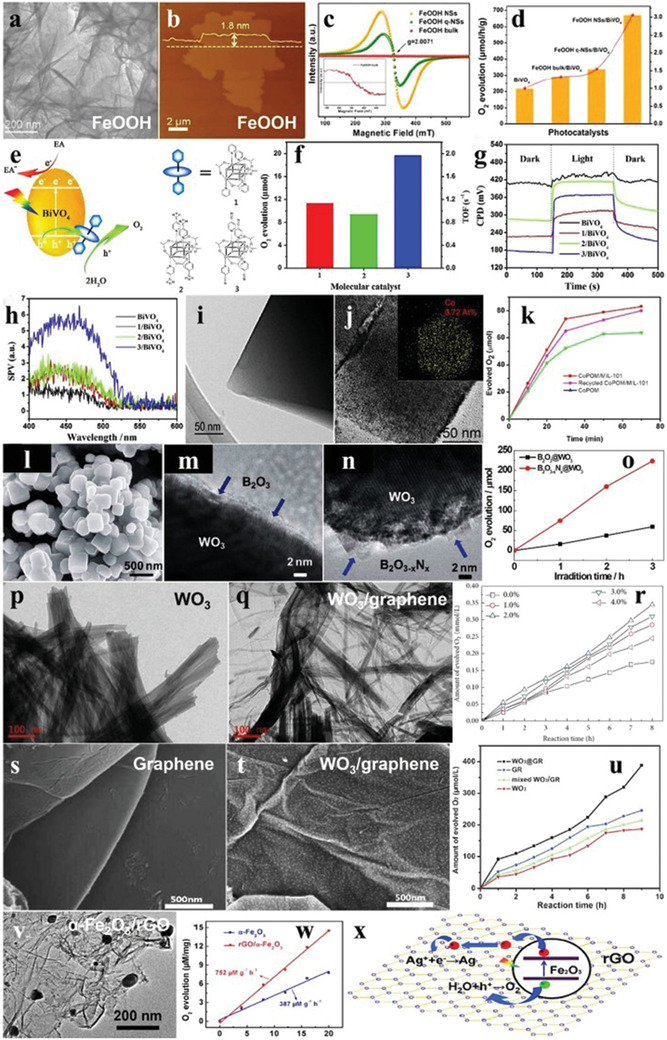
a) TEM image and b) AFM image of ultrathin FeOOH nanosheets. c) ESR spectra of FeOOH with distinct morphologies. d) Time courses of oxidation evolution for pristine BiVO_4_ and various kinds of BiVO_4_/FeOOH materials. Reproduced with permission.^[^ ^143^ ^]^ Copyright 2019, Royal Society of Chemistry. e) Schematic diagram of reaction mechanism and f) the O_2_ evolution production of BiVO_4_ loaded with cobalt‐based compounds. g) Kelvin probe force microscopy and h) surface photovoltage of pristine BiVO_4_ and cobalt‐based compound‐loaded samples. Reproduced with permission.^[^ ^144^ ^]^ Copyright 2016, Elsevier. i) TEM image of MIL‐101. j) TEM image and EDX mapping image (inset) of Co element of CoPOM encapsulated in MIL‐101. k) Time courses of oxidation evolution for pristine CoPOM, CoPOM/MIL‐101, and recycled CoPOM/MIL‐101. Reproduced with permission.^[^ ^145^ ^]^ Copyright 2015, Elsevier. l) SEM image of WO_3_ loaded with B_2_O_3_. HRTEM images of WO_3_ loaded with m) B_2_O_3_ and n) B_2_O_3−_ *_x_*N*_x_*. o) Time courses of oxidation evolution for B_2_O_3_‐loaded WO_3_ and B_2_O_3−_ *_x_*N*_x_*‐loaded WO_3_. Reproduced with permission.^[^ ^146^ ^]^ Copyright 2012, Royal Society of Chemistry. p,q) TEM images of WO_3_ nanowires and WO_3_/graphene nanowires. r) Time courses of oxidation evolution for WO_3_ nanowires and WO_3_/graphene nanowires. Reproduced with permission.^[^ ^147^ ^]^ Copyright 2014, Hindawi Publishing Corporation. SEM images of s) graphene and t) WO_3_/graphene. u) Time courses of oxidation evolution for graphene, WO_3_, WO_3_/graphene, and physical mixed WO_3_/graphene. Reproduced with permission.^[^ ^148^ ^]^ Copyright 2012, Royal Society of Chemistry. v) TEM image of *α*‐Fe_2_O_3_/rGO. w) Time courses of oxidation evolution for pristine *α*‐Fe_2_O_3_ and *α*‐Fe_2_O_3_/rGO. x) Schematic diagram of reaction mechanism of *α*‐Fe_2_O_3_/rGO. Reproduced with permission.^[^ ^149^ ^]^ Copyright 2013, American Chemical Society.

As one of the most abundant metals on earth, Fe species as cocatalysts have a great potential in the substitution of the traditional noble metal oxides. Due to the poor conductivity of Fe(oxy)hydroxides, 2D ultrathin FeOOH nanosheets with a thickness of ≈1.8 nm were prepared by a bottom‐up strategy, which had abundant OVs compared to the bulk FeOOH and FeOOH nanoparticles (**Figure** **20**a–c). The FeOOH nanosheets can be easily loaded on BiVO_4_ by electrostatic interaction, leading to the O_2_ evolution rate of 67.2 µmol h^−1^ with 0.5% loading amount of FeOOH nanosheets (Figure 20d).^[^ ^143^ ^]^

In addition to the TMOs as cocatalysts, transition metal complexes also play an important role in promoting the water oxidation activity of photocatalysts. BiVO_4_ loaded with three kinds of cobalt‐based complexes (Co_4_O_4_(O_2_CMe)_4_L_4_, L = py, Mepy, and CNpy, denoted as 1–3, respectively; Figure 20e) were prepared by heating and refluxing routes, among which the BiVO_4_ loaded with No. 3 cobalt‐based molecular catalyst exhibited an excellent photocatalytic activity with a high TOF of 2 s^−1^ (Figure 20f). Kelvin probe force microscopy (KPFM) and surface photovoltage (SPV) measurements revealed that the ΔCPD signal (reflecting the migration extent of photogenerated holes to the surface of photocatalysts) and SPV amplitude were enhanced for BiVO_4_/Co_4_O_4_(O_2_CMe)_4_L_4_ (Figure 20g,h), indicating that the separation efficiency of photogenerated carriers of BiVO_4_ was improved by loading of cobalt‐based complexes.^[^ ^144^ ^]^

Due to the stable structure, the MOF materials can also be used as the supports of photocatalysts to promote the O_2_ evolution. To improve the stability and photocatalytic activity of the polyoxometalates [Co(H_2_O)_2_(PW_9_O_34_)_2_]^10−^ (denoted as CoPOM), the MOF MIL‐101 was applied as the support to encapsulate CoPOM inside the cavity. TEM and energy dispersive X‐ray (EDX) mapping images (Figure 20i,j) showed that the CoPOM nanoparticles were evenly distributed in the channel of MIL‐101 by ion exchange approach. After being immobilized inside the cavity of MIL‐101, the O_2_ evolution production of the composite was higher than that of pristine CoPOM, and the slight decrease in O_2_ evolution activity after one circle reaction was ascribed to the leakage of CoPOM within MIL‐101 (Figure 20k).^[^ ^145^ ^]^

Compared with traditional metal‐based cocatalysts, the nonmetallic oxide cocatalysts can be modified by the heteroatoms, such as nitrogen doping. Through calcination under ammonia atmosphere, WO_3_ loaded with B_2_O_3−_ *_x_*N*_x_* nanoclusters was synthesized. TEM images (Figure 20m,n) showed obvious nanocluster layers of both WO_3_/B_2_O_3_ and WO_3_/B_2_O_3−_ *_x_*N*_x_*. The O_2_ production amount of WO_3_/B_2_O_3−_ *_x_*N*_x_* was higher than WO_3_/B_2_O_3_ (Figure 20o), demonstrating positive effect of B_2_O_3−_ *_x_*N*_x_* in promoting the O_2_ evolution of WO_3_ than B_2_O_3_. It provided an insight for the exploration of nonmetallic cocatalysts.^[^ ^146^ ^]^ Another widely used 2D cocatalyst is graphene, which can act as the carrier of photocatalysts to promote the separation of photogenerated carriers owing to the excellent conductivity. In Figure 20p–r, the WO_3_/graphene nanowires with different mass ratios of graphene that were prepared by a facile hydrothermal route displayed higher O_2_ evolution performance than the WO_3_ nanowires, because graphene promoted the separation efficiency of charge carriers by accepting the photogenerated electrons from WO_3_.^[^ ^147^ ^]^ Due to the existence of the low resistance conduction path, the WO_3_/graphene with the particle diameter of ≈12 nm exhibited higher O_2_ evolution production than bare WO_3_ and physically mixed WO_3_ and graphene (Figure 20s–u).^[^ ^148^ ^]^ Similarly, the photocatalytic activity of *α*‐Fe_2_O_3_ was also elevated by coupling with reduced graphene oxide (rGO) as the electron transfer platform (Figure 20v–x).^[^ ^149^ ^]^

### 3.2 Heterojunction Construction

Two crucial factors that hinder the photocatalytic performance of semiconductor photocatalysts are the insufficient photoabsorption and the rapid recombination of photogenerated electron and holes during their migration from the bulk to the surface of photocatalysts. By combining two or more photocatalysts with the suitable band structures to form the II‐type or Z scheme heterojunction, the weak photoabsorption of wide‐bandgap semiconductors can be compensated by narrow‐bandgap semiconductor. Moreover, the photogenerated electrons and holes can be effectively separated between different components on the basis of their band structures, collectively resulting in enhanced photocatalytic O_2_ evolution activity.

#### 3.2.1 II‐type Heterojunction

**Figure 21:**
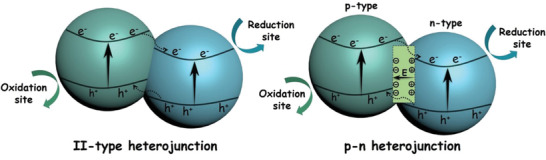
Schematic diagram of II‐type heterojunction and p–n heterojunction.

II‐type heterojunction is formed by two or more semiconductors with staggered band structures. The energy potential difference can propel the photogenerated electrons and holes to migrate to the semiconductors with more positive CB and more negative VB, respectively. II‐type heterojunction is a p–n junction if it consists of a p‐type semiconductor and an n‐type semiconductor. The as‐formed built‐in electric field in the p–n junction will further boost the directional transfer of photogenerated carriers of the photocatalysts.^[^ ^150^, ^151^, ^152^ ^]^ The schematic diagrams of II‐type heterojunction and p–n heterojunction are shown in **Figure** **21**.

**Figure 22:**
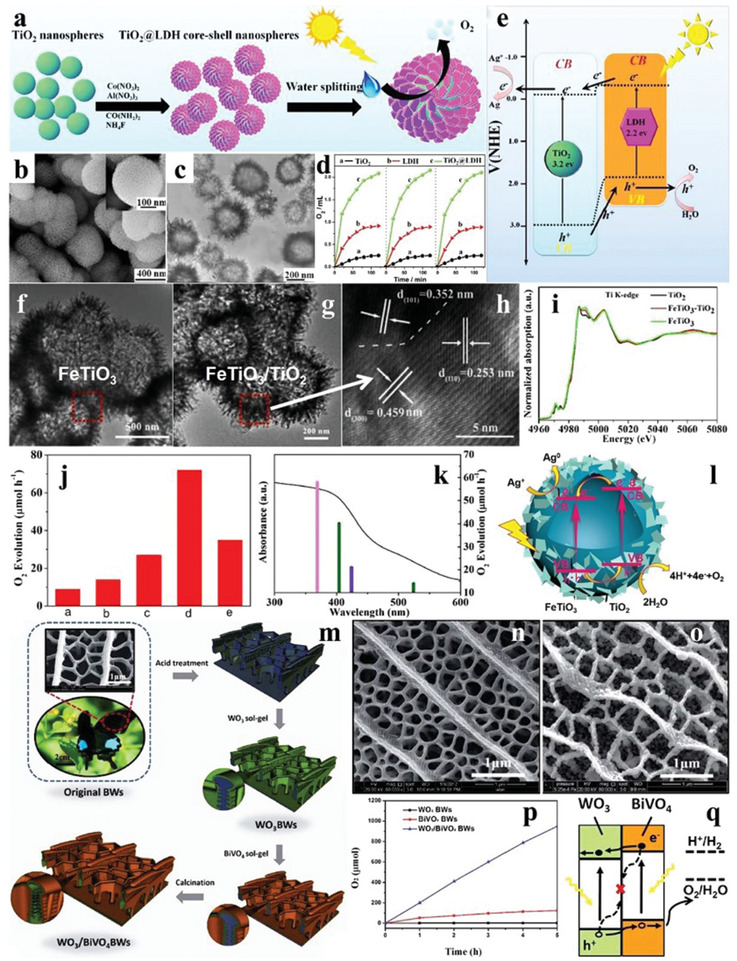
a) Schematic diagram of the synthesis and O_2_ evolution of TiO_2_@CoAl‐LDH hollow nanospheres. b) SEM image and c) TEM image of TiO_2_@CoAl‐LDH hollow nanospheres. d) Time courses of oxidation evolution along with three cycling tests for TiO_2_, CoAl‐LDH, and TiO_2_@CoAl‐LDH hollow nanospheres. e) Schematic diagram of reaction mechanism of TiO_2_@CoAl‐LDH hollow nanospheres. Reproduced with permission.^[^ ^153^ ^]^ Copyright 2015, Wiley. f) TEM image of FeTiO_3_. g,h) TEM and HRTEM image of FeTiO_3_–TiO_2_ hollow spheres. i) The X‐ray absorption near‐edge spectra of Ti K‐edge of TiO_2_, FeTiO_3_, and FeTiO_3_–TiO_2_. j) The O_2_ evolution rates of pristine TiO_2_ and FeTiO_3_–TiO_2_ hollow spheres with mass ratios of 1:0.25, 1:0.5, 1:0.75, and 1:1. k) UV–vis spectrum and wavelength‐dependent O_2_ evolution rates of FeTiO_3_–TiO_2_ hollow spheres. l) Schematic diagram of reaction mechanism of FeTiO_3_–TiO_2_ hollow spheres. Reproduced with permission.^[^ ^154^ ^]^ Copyright 2015, Royal Society of Chemistry. m) Schematic diagram of synthesis of WO_3_/BiVO_4_. SEM images of n) pristine WO_3_ and o) WO_3_/BiVO_4_. p) Time courses of oxidation evolution for WO_3_, BiVO_4_, and WO_3_/BiVO_4_. q) Schematic diagram of reaction mechanism of WO_3_/BiVO_4_. Reproduced with permission.^[^ ^155^ ^]^ Copyright 2017, Royal Society of Chemistry.

Through a facile in situ crystallization, the CoAl‐layered double hydroxide (CoAl‐LDH) was grown on the surface of TiO_2_ hollow nanospheres (TiO_2_@CoAl‐LDH) to construct TiO_2_@CoAl‐LDH hollow nanospheres with average size of ≈250 nm, which achieved the utilization of the full sunlight spectrum (**Figure** **22**a–c). Under the irradiation of simulated sunlight, the O_2_ evolution rate of TiO_2_@CoAl‐LDH (2.34 mmol h^−1^ g^−1^) was much higher than that of the pristine TiO_2_ hollow nanospheres (0.27 mmol h^−1^ g^−1^) (Figure 22d). The DOS calculations revealed that the coupling of TiO_2_ and CoAl‐LDH made the CB potential of TiO_2_ decreased and bandgap of CoAl‐LDH narrowed at the same time, thus the photogenerated electrons can migrate from the CB of CoAl‐LDH to that CB of TiO_2_, while holes from VB of TiO_2_ were injected into that of CoAl‐LDH, leading to the effective separation of charge carriers (Figure 22e).^[^ ^153^ ^]^ The FeTiO_3_–TiO_2_ porous hollow architectures with the interconnected nanosheets anchored on the surface were synthesized by a two‐step solvothermal method followed by the calcination treatment (Figure 22g,h). X‐ray absorption near‐edge spectra (XANES) of Ti K‐edge revealed that the FeTiO_3_–TiO_2_ and pristine component had the similar local symmetry (Figure 22i). When the atomic ratio of Ti to Fe was 0.75, the O_2_ evolution rate of FeTiO_3_–TiO_2_ hollow sphere reached the maximum, ≈2 times that of pristine FeTiO_3_ (Figure 22j). The improved O_2_ evolution performance was attributed to the formation of the II‐type heterojunction between FeTiO_3_ and TiO_2_, which promoted the separation of photogenerated charge carriers (Figure 22l).^[^ ^154^ ^]^ Interestingly, inspired by the architecture of butterfly wings in nature, 3D WO_3_/BiVO_4_ heterojunction was fabricated by a facile one‐step sol–gel route with *Paris papilio* as the biological template (Figure 22m). As shown in Figure 22n,o, both the as‐prepared WO_3_ and WO_3_/BiVO_4_ photocatalysts retained the quasi‐honeycomb morphology after the calcination treatment. The O_2_ yield of WO_3_/BiVO_4_ was 950 µmol after 5 h irradiation, which was 7.6‐fold higher than that of pristine BiVO_4_ (Figure 22p). The synergetic effects of the porous quasi‐honeycomb structure and the formed II‐type heterojunction between WO_3_ and BiVO_4_ that boosted the visible light absorption and photogenerated charge separation, thereby contributing to the increased photocatalytic O_2_ evolution production (Figure 22q).^[^ ^155^ ^]^

**Figure 23:**
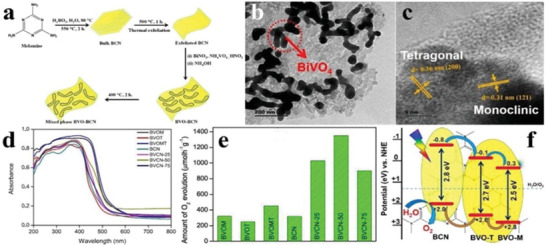
a) Schematic diagram of synthesis of BiVO_4_‐B‐doped g‐C_3_N_4_. b) TEM and c) HRTEM image of BiVO_4_‐B‐doped g‐C_3_N_4_. d) DRS of varieties of samples. e) The O_2_ evolution rates of varieties of samples. f) Schematic diagram of reaction mechanism of BiVO_4_‐B‐doped g‐C_3_N_4_. Reproduced with permission.^[^ ^156^ ^]^ Copyright 2019, American Chemical Society.

Through a facile impregnation route followed by calcination treatment, the B‐doped g‐C_3_N_4_ was deposited on the dual‐phases BiVO_4_ (monoclinic and tetragonal phases, denoted as BVOMT) to form the p–n heterojunction (**Figure** **23**a). TEM and high‐resolution TEM (HRTEM) images demonstrated that the surface of g‐C_3_N_4_ nanosheets was decorated with BiVO_4_ cashew nut (Figure 23b,c). The BVOMT showed higher O_2_ evolution rate (452.8 µmol h^−1^ g^−1^) than the monoclinic‐phase BiVO_4_ (321 µmol h^−1^ g^−1^) and tetragonal‐phase BiVO_4_ (256 µmol h^−1^ g^−1^) (Figure 23d,e). Furthermore, when BVOMT was combined with B‐doped g‐C_3_N_4_, the as‐synthesized BVCN‐50 with the strongest visible light absorption exhibited the highest O_2_ evolution rate (1027.2 µmol h^−1^ g^−1^), which was attributed to the formation of p–n heterojunction (Figure 23f).^[^ ^156^ ^]^

#### 3.2.2 Z‐Scheme Heterojunction

**Figure 24:**
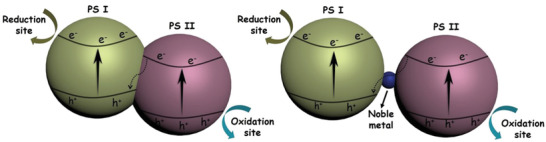
Schematic diagram of Z‐scheme heterojunction without (left) and with (right) mediators.

Although the II‐type/p–n heterojunction can effectively improve the separation efficiency of the photogenerated charge carriers, the photogenerated carriers are all transferred to lower energy levels, which reduces the redox driving force of photocatalysts.^[^ ^157^, ^158^ ^]^ As a result, the concept of Z‐scheme has been proposed by recombination of the photogenerated holes of PS I and the electrons of PS II (**Figure** **24**). The advantage of the Z‐scheme heterojunction is that it can spontaneously promote the separation of photogenerated carriers and maintain the strong redox abilities of photocatalysts, which has attracted enormous attentions in photocatalytic water splitting in recent years.^[^ ^159^ ^]^

**Figure 25:**
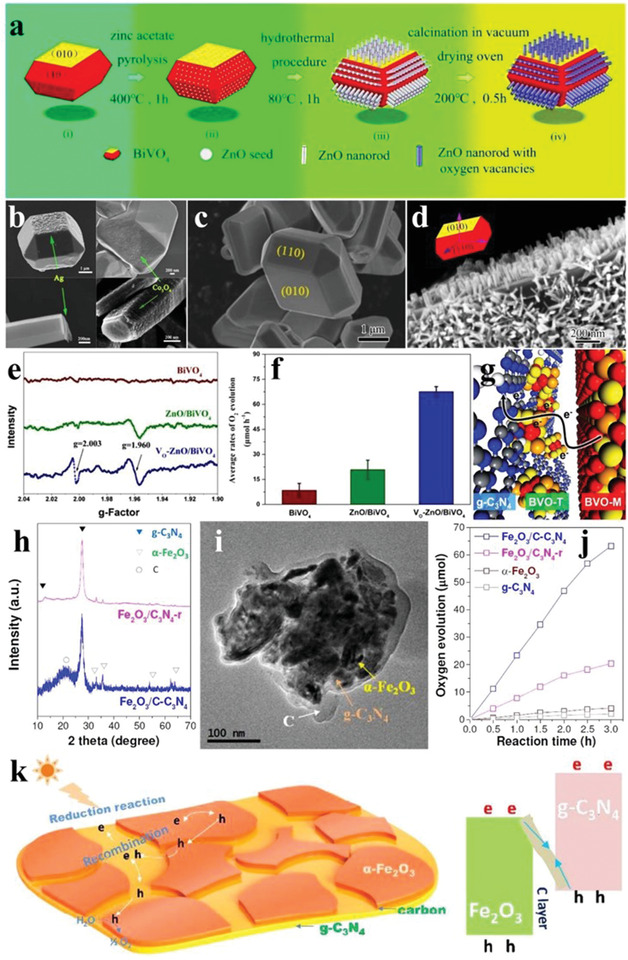
a) Schematic diagram of synthesis of ZnO/BiVO_4_. b) SEM images of BiVO_4_ and ZnO photodeposited with Ag and Co_3_O_4_. SEM images of c) BiVO_4_ and d) ZnO/BiVO_4_. e) ESR spectra of varieties of samples. f) The O_2_ evolution rates of varieties of samples. Reproduced with permission.^[^ ^160^ ^]^ Copyright 2018, Elsevier. g) Schematic diagram of water oxidation of mixed‐phase BiVO_4_/g‐C_3_N_4_, BVO‐T, and BVO‐M are represented the monoclinic scheelite and tetragonal zircon phase, respectively. Reproduced with permission.^[^ ^161^ ^]^ Copyright 2019, Royal Society of Chemistry. h) XRD patterns of *α*‐Fe_2_O_3_/C_3_N_4_‐r and *α*‐Fe_2_O_3_/C–g‐C_3_N_4_. i) TEM image of *α*‐Fe_2_O_3_/C‐C_3_N_4_. j) The O_2_ evolution production of varieties of samples. k) Schematic diagram of reaction mechanism of *α*‐Fe_2_O_3_/C‐C_3_N_4_. Reproduced with permission.^[^ ^162^ ^]^ Copyright 2018, American Chemical Society.

The photodeposition experiment revealed that the {010} and {110} facets of BiVO_4_ were the reduction and oxidation facets, respectively (**Figure** **25**b), while the endpoint and surface of ZnO nanorods were the accumulation regions of electrons and holes, respectively. To probe the charge separation and migration behavior between ZnO and BiVO_4_, the 1D/3D ZnO/BiVO_4_ was assembled by hydrothermal route followed by calcination treatment (Figure 25a). SEM images and electron paramagnetic resonance (EPR) spectra confirmed that the OV‐rich ZnO nanorods were vertically anchored on the surface of BiVO_4_ (Figure 25c–e). Under visible light illumination, the O_2_ evolution rate of ZnO/BiVO_4_ (68 µmol h^−1^) was much higher than that of the pristine BiVO_4_ (Figure 25f). It was demonstrated that the photogenerated electrons and holes were separately gathered on {010} and {110} facets of BiVO_4_ driven by the internal electric field. Partial holes on {110} facets were recombined with the electrons that enriched on the endpoints of ZnO nanorods to form a Z‐scheme heterojunction, which led to efficient charge separation.^[^ ^160^ ^]^ Besides, the mixed‐phase BiVO_4_ (monoclinic and tetragonal phases) were applied as the bridge to construct BiVO_4_/g‐C_3_N_4_ Z‐scheme junction for accelerating the charge separation and water oxidation (Figure 25g).^[^ ^161^ ^]^ To realize the tight coupling between *α*‐Fe_2_O_3_ and g‐C_3_N_4_, an SSR route was carried out to synthesize the 2D/2D *α*‐Fe_2_O_3_/C–g‐C_3_N_4_ (C–g‐C_3_N_4_ indicates g‐C_3_N_4_ with amorphous carbon). The weaker peak in XRD patterns of *α*‐Fe_2_O_3_/C‐C_3_N_4_ attributed to the exfoliation and partial carbonization of g‐C_3_N_4_ after calcination (Figure 25h), as confirmed by the obvious amorphous carbon around the edges of g‐C_3_N_4_ (Figure 25i). Owing to the formed Z‐scheme junction between *α*‐Fe_2_O_3_ and C–g‐C_3_N_4_, the O_2_ evolution production rate of *α*‐Fe_2_O_3_/C–g‐C_3_N_4_ (22.3 µmol h^−1^) was 30‐fold higher than that of the pristine g‐C_3_N_4_ (0.7 µmol h^−1^) (Figure 25j,k).^[^ ^162^ ^]^

**Figure 26:**
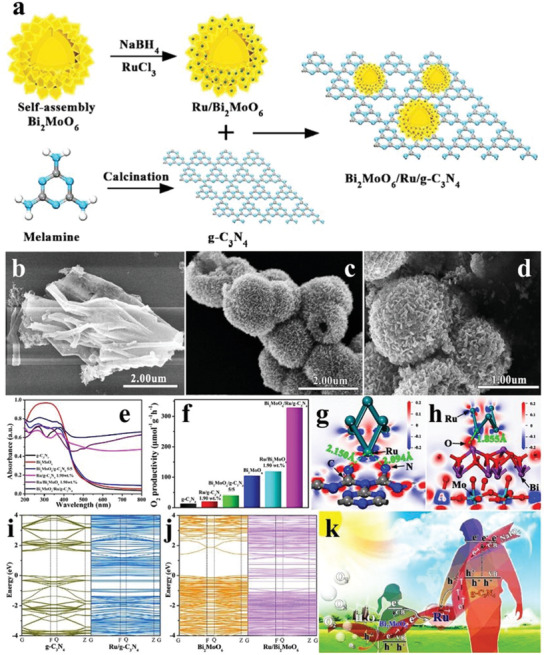
a) Schematic diagram of synthesis of Bi_2_MoO_6_/Ru/g‐C_3_N_4_. SEM images of b) g‐C_3_N_4_, c) Bi_2_MoO_6_, and d) Bi_2_MoO_6_/Ru/g‐C_3_N_4_. e) DRS of varieties of samples. f) The O_2_ evolution rates of varieties of samples. Charge density difference of g) Ru/g‐C_3_N_4_ and h) Ru/Bi_2_MoO_6_. Band structures of i) g‐C_3_N_4_ and Ru/g‐C_3_N_4_, and j) Bi_2_MoO_6_ and Ru/Bi_2_MoO_6_. k) Schematic diagram of reaction mechanism of Bi_2_MoO_6_/Ru/g‐C_3_N_4_. Reproduced with permission.^[^ ^167^ ^]^ Copyright 2019, American Chemical Society.

As a layered perovskite, Bi_2_MoO_6_ has a great potential in the field of photocatalysis due to the layered crystal structure and suitable bandgap. The heterojunction of Bi_2_MoO_6_ hybridized with the good electron transporters, such as g‐C_3_N_4_, has shown excellent photocatalytic performances in terms of the degradation of organic pollutants, water splitting for H_2_ evolution, etc.^[^ ^163^, ^164^, ^165^, ^166^ ^]^ To further improve the activity or stability, it is significant to introduce appropriate electron transfer media, like Au, Rh, and Ru. Recently, the Bi_2_MoO_6_/Ru/g‐C_3_N_4_ catalyst was fabricated by the solvothermal method combined with the reduction of precursor (**Figure** **26**a,d). DRS indicated that the incorporated Ru endowed the material with the absorption in the entire visible light region (Figure 26e). The calculation results of charge density difference and band structure manifested the formation of chemical bonds between Ru and O atoms of Bi_2_MoO_6_ (Figure 26g–j). Under visible light irradiation, Bi_2_MoO_6_/Ru/g‐C_3_N_4_ exhibited the highest O_2_ evolution rate of 328.34 µmol h^−1^ g^−1^, which was ≈3 and 25 times that of Bi_2_MoO_6_ and g‐C_3_N_4_, respectively (Figure 26f). It was attributed to that the metallic Ru as the electron transfer media promoted the recombination of photogenerated electrons from Bi_2_MoO_6_ and holes from g‐C_3_N_4_, allowing Bi_2_MoO_6_ to retain the strong oxidizing capability (Figure 26k).^[^ ^167^ ^]^

### 3.3 Doping and Vacancy Formation

Doping or vacancy creation can break the periodicity of crystal atomic arrangement and induce the lattice distortion, which lead to the formation of impurity states or defect states in the forbidden band, thus extending the light response range.^[^ ^168^ ^]^ Besides, they can also promote the separation of photogenerated charge carriers, enhancing the photocatalytic O_2_ evolution performance.

#### 3.3.1 Doping

Since 1982, it has been found that the incorporation of a certain amount of transition metals, such as Cr and Ru, into TiO_2_ allows it to absorb more visible light. After that, metallic doping was employed to improve the photocatalytic activity of semiconductors.^[^ ^169^ ^]^ At the beginning of this century, nonmetallic elements doping (e.g., B, N) has gradually become the mainstream, as they bring more flexible tunability in the improvement of photocatalytic activity.^[^ ^170^, ^171^ ^]^ Nowadays, a generally accepted viewpoint is that the effect of metallic/nonmetallic doping for photocatalysts can be affected by the types of element, doping methods, concentration of dopant, and doping position.

**Figure 27:**
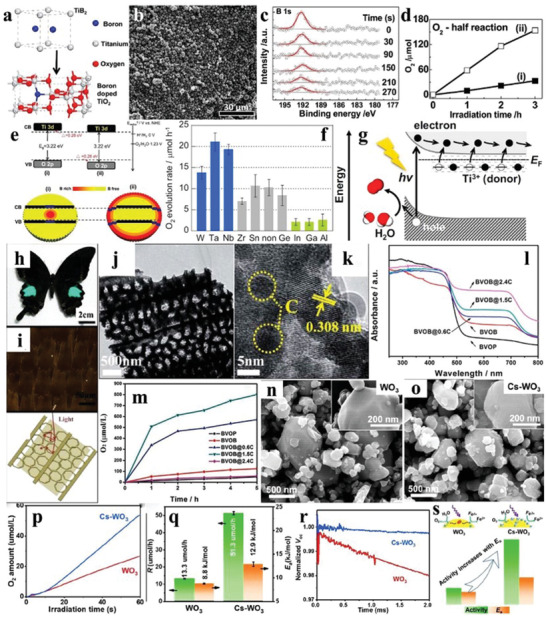
a) Schematic diagram of crystal structures of TiB_2_ and anatase TiO_2_. b) SEM image of TiO_2_ microspheres. c) Argon ion sputtering dependent XPS spectra of B 1s of TiO_2_ microspheres. d) Time courses of oxidation evolution for TiO_2_ microspheres i) before and ii) after heat treatment. e) Schematic diagram of boron distribution and electronic structure inside TiO_2_ microspheres before and after heat treatment. Reproduced with permission.^[^ ^172^ ^]^ Copyright 2012, Wiley. f) The O_2_ evolution rates of pristine rutile TiO_2_ and TiO_2_ doped with various kinds of metallic cations. g) Schematic diagram of reaction mechanism of TiO_2_ doped with metallic cations. Reproduced with permission.^[^ ^173^ ^]^ Copyright 2018, Elsevier. h) Digital picture, and i) optical microscopy image and model of butterfly wings. j,k) TEM and HRTEM image of BiVO_4_ doped with 1.5 wt% C. l) DRS and m) time courses of oxidation evolution for pristine BiVO_4_ and BiVO_4_ doped with various amounts of C. Reproduced with permission.^[^ ^174^ ^]^ Copyright 2013, Royal Society of Chemistry. SEM images and enlarged views (inset) of n) WO_3_ and o) Cs–WO_3_. p) Time courses of oxidation evolution, and q) the O_2_ evolution rates and energy barrier of WO_3_ and Cs–WO_3_. r) Open circuit voltage decay of WO_3_ and Cs–WO_3_. s) Schematic diagram of reaction mechanism of WO_3_ and Cs–WO_3_. Reproduced with permission.^[^ ^175^ ^]^ Copyright 2019, American Chemical Society.

Based on the mature research works of elemental doping in TiO_2_, the concept of gradient doping with nonmetallic heteroatoms in TiO_2_ to improve the electronic structure has been proposed. As shown in **Figure** **27**a,b, the anatase TiO_2_ microspheres doped with boron were synthesized by hydrothermal method and heat treatment with TiB_2_ as the precursor of titanium and boron. The X‐ray photoelectron spectroscopy (XPS) spectra with argon ion sputtering revealed that the binding energy of B 1s in the TiO_2_ microspheres changed from 187.9 to 192.2 eV, indicating the transition of substitutional boron (B*^*δ*^* ^−^) to interstitial boron (B*^*σ*^* ^+^) during the heat treatment process (Figure 27c). It was found that the O_2_ evolution of TiO_2_ microspheres after thermal treatment was 4.5 times that of previous one (Figure 27d), which was attributed to the diffusion of boron from the core to the edges of microspheres, making the VB of TiO_2_ shifted to a more positive energy level with stronger oxidation ability (Figure 27e).^[^ ^172^ ^]^ Since the effect of doping can be affected by the types of element, it is interesting to investigate the influence of the valence state of doped elements in photocatalysts. As shown in Figure 27f, the O_2_ evolution rates of TiO_2_ doped with W^6+^, Ta^5+^, or Nb^5+^ were higher than that of the pristine TiO_2_, while doping of Zr^4+^, Sn^4+^, or Ge^4+^ had little effect on the photocatalytic activity of TiO_2_. In contrast, the O_2_ evolution performance of TiO_2_ was reduced by doping In^3+^, Ga^3+^, or Al^3+^. According to the classical Kröger–Vink theory, when the metallic cations with a higher valence than Ti^4+^ were doped into the TiO_2_ lattice (denoted as donor doping), the electron will increase in concentration and be captured into the Ti^4+^ lattice, tuning the latter into Ti^3+^ species. Then, the Fermi energy level was shifted upward and a built‐in electric field was formed, promoting the separation of photogenerated charge carriers (Figure 27g).^[^ ^173^ ^]^

The butterfly wings in nature are composed of uniformly arranged structures, in which the morphology of porous honeycomb endows them with the strong absorbance for the external sunlight (Figure 27h,i). Inspired by this architecture, the C‐doped BiVO_4_ was synthesized by the sol–gel route followed by subsequent thermal treatment with butterfly wings as the sacrificial template. As shown in Figure 27j,k, the original porous honeycomb structure was basically retained for the as‐synthesized material and the lattice spacing of 0.308 nm was assigned to the {121} facets of BiVO_4_. DRS revealed that the incorporation of C allowed BiVO_4_ stronger absorption intensity in visible light region (Figure 27l). When the temperature of thermal treatment and doping amount of C was 400 °C and 1.5 wt%, respectively, the sample showed the highest O_2_ evolution activity (800 µmol L^−1^; Figure 27m), which was ascribed to the synergistic effect of the unique structure and C doping, resulting in enhanced absorption of visible light and separation of the carriers.^[^ ^174^ ^]^

In order to investigate the main factor that hinder the photocatalytic water oxidation, WO_3_ treated with cesium (denoted as Cs–WO_3_) was synthesized by the impregnation approach. After the introduction of cesium, an amorphous layer appeared on the surface of Cs–WO_3_ with slightly changed size (Figure 27n,o). Compared to pristine WO_3_, the O_2_ evolution rate of Cs–WO_3_ increased by three times (51.3 µmol h^−1^) (Figure 27p). Contrary to previous view that the lower energy barrier was beneficial to the photocatalytic reaction, Cs–WO_3_ with a higher energy barrier had a longer photogenerated carrier lifetime than that of WO_3_ as revealed by the open circuit voltage decay curves (Figure 27q,r). Based on these advances, Li's group proposed that the main bottleneck for O_2_ evolution is to enrich the long‐lived photogenerated holes that were involved in the oxidation reaction on the surface of photocatalysts (Figure 27s),^[^ ^175^ ^]^ as widely supported by other works.^[^ ^176^, ^177^ ^]^

**Figure 28:**
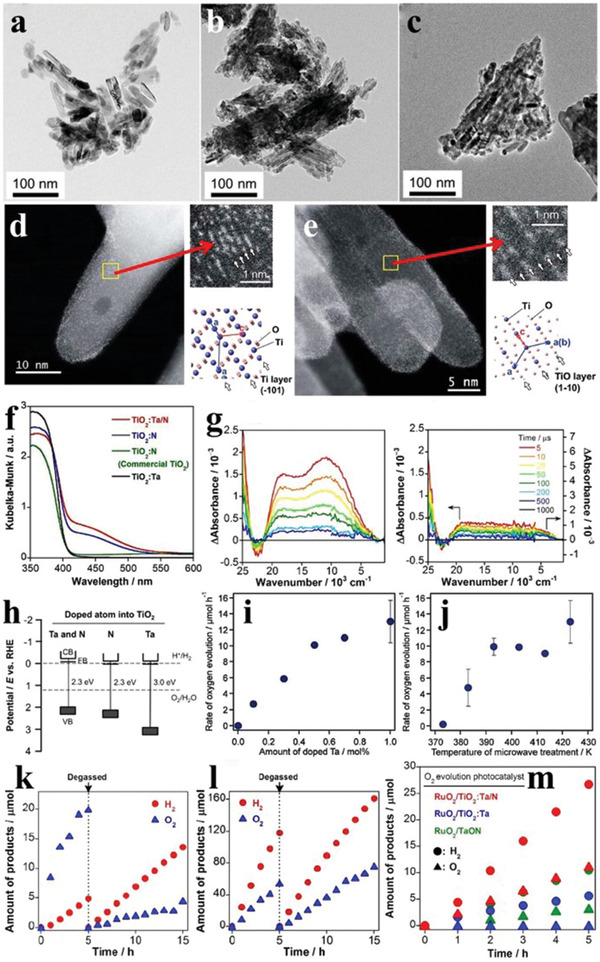
TEM images of a) pristine rutile TiO_2_, and b) TiO_2_ doped with Ta and c) codoped with Ta/N. Reproduced with permission.^[^ ^179^ ^]^ Copyright 2017, Royal Society of Chemistry. HAADF‐STEM images, enlarged views, and crystal structures of TiO_2_ codoped with Ta/N in d) [121] and e) [111] directions. Reproduced with permission.^[^ ^180^ ^]^ Copyright 2019, Royal Society of Chemistry. f) DRS of pristine TiO_2_ and varieties of samples. g) Transient absorption spectra of TiO_2_ doped with N and codoped with Ta/N. h) Schematic diagram of band positions of TiO_2_ doped with N, Ta, and codoped with Ta/N. Reproduced with permission.^[^ ^179^ ^]^ Copyright 2017, Royal Society of Chemistry. The O_2_ evolution rates of TiO_2_ codoped with Ta/N as a function of i) doping amounts of Ta species and j) temperatures. Reproduced with permission.^[^ ^180^ ^]^ Copyright 2019, Royal Society of Chemistry. Time courses of overall water splitting evolution for RuO_2_/Ta–N codoped TiO_2_ combined with RuO_2_/Rh‐doped SrTiO_3_ in k) NaIO_3_ aqueous solution and l) FeCl_3_ aqueous solution. m) Time courses of overall water splitting evolution for varieties of O_2_ evolution photocatalysts combined with RuO_2_/Rh‐doped SrTiO_3_ in FeCl_3_ aqueous solution. Reproduced with permission.^[^ ^179^ ^]^ Copyright 2017, Royal Society of Chemistry.

The band position of TiO_2_ can be adjusted through the nitridation, and its water oxidation performance can be improved by the donor doping,^[^ ^173^, ^178^ ^]^ thus the synergistic effect of metal and nonmetal codoping on the water oxidation activity of rutile TiO_2_ was investigated. Kazuhiko's group reported that the rutile TiO_2_ nanorods codoped with Ta/N species (denoted as TiO_2_:Ta/N) were synthesized by the microwave‐assisted hydrothermal route followed by the subsequent calcination treatment in the ammonia atmosphere (**Figure** **28**a–c). The doping sites of Ta^5+^ within the crystal of TiO_2_ were mainly along the [101] and [110] directions (Figure 28d,e). DRS and schematic diagram of band positions in Figure 28f,h revealed that the increased absorbance ability of visible light for TiO_2_:Ta/N was ascribed to the doping of N species, while the doping of single Ta species enlarged the bandgap of TiO_2_. The transient absorption spectra indicated that the as‐synthesized TiO_2_:Ta/N showed a much lower concentration of deeply trapped charge carriers compared with N‐doped TiO_2_, implying a higher photocatalytic activity of TiO_2_:Ta/N (Figure 28g). Based on above results, it can be proclaimed that the role of Ta doping is to improve the separation efficiency of photogenerated carriers of TiO_2_, while the incorporation of N species endows TiO_2_ with stronger visible light absorbance. Additionally, the O_2_ evolution rate rose with increasing the doping amount of Ta species and the temperature of microwave‐assisted treatment, indicating that the photocatalytic activity of rutile TiO_2_ was affected by both the doping level and the crystallinity of the catalysts (Figure 28i,j). Importantly, the photocatalytic activity of OWS was closely related to the types of redox cycle mediator and the kinds of O_2_ evolution photocatalyst. It was revealed that the TiO_2_–SrTiO_3_ Z‐scheme system exhibited a higher catalytic activity with Fe^3+^/Fe^2+^ as the redox cycle mediator than that with IO_3_^−^/I^−^ because the Fe^2+^ electron donor has a stronger capability in hindering the backward reaction of SrTiO_3_ (Figure 28k,l). Furthermore, the TiO_2_:Ta/N showed the highest OWS performance (Figure 28m), and a solar‐to‐hydrogen conversion efficiency (STH) of 0.021% was obtained with Fe^3+^/Fe^2+^ as the redox cycle mediator, and a higher STH of 0.039% was achieved with TiO_2_:Ta/N (IrO_2_ as the cocatalyst) as the O_2_ evolution component in the Rh‐doped SrTiO_3_‐based Z‐scheme system.^[^ ^179^, ^180^ ^]^

#### 3.3.2 Vacancy Formation

In addition to doping, another strategy for improving the O_2_ evolution performance based on defect modulation is the vacancy creation. Vacancy is one of the intrinsic defects, which can introduce the defect energy level into the forbidden band of semiconductors, thereby enhancing the light absorbance ability and charge separation.^[^ ^181^, ^182^, ^183^, ^184^, ^185^ ^]^

**Figure 29:**
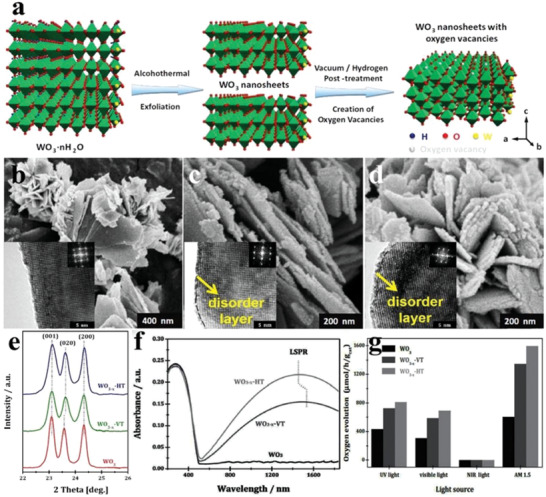
a) Schematic diagram of synthesis of WO_3_ nanosheets with OVs. SEM images and HRTEM images (inset) of b) pristine WO_3_ nanosheets, c) WO_3_ nanosheets calcined in H_2_ atmosphere, and d) vacuum. e) Enlarged view of XRD patterns of pristine WO_3_ nanosheets, and WO_3_ nanosheets calcined in H_2_ atmosphere and vacuum. f) DRS of pristine WO_3_ nanosheets, and WO_3_ nanosheets calcined in H_2_ atmosphere and vacuum. g) The O_2_ evolution rates of pristine WO_3_ nanosheets, and WO_3_ nanosheets calcined in H_2_ atmosphere and vacuum with varieties of irradiation conditions. Reproduced with permission.^[^ ^188^ ^]^ Copyright 2015, Wiley.

OVs are one of the most common vacancies, and the construction of surface OVs is more beneficial to the separation of photogenerated carriers than the OVs in the bulk.^[^ ^186^ ^]^ In recent years, 2D monocrystalline nanosheets are the ideal candidates to create OVs. For instance, the OVs were in situ generated on the surface of BiOCl single‐crystalline nanosheets with the exposed {010}/{001} facets by the hydrothermal method followed by UV‐light irradiation. The water molecules adsorbed over OVs on the {010} facets in the dissociated manner were more easily to be oxidized than that on {001} facets in the molecular manner, thus leading to an enhanced O_2_ production activity.^[^ ^187^ ^]^ Yan et al. fabricated WO_3_ single‐crystalline nanosheets with OVs introduced by the exfoliation and the following calcination treatment (**Figure** **29**a). Compared to pristine WO_3_ nanosheets, the morphology was hardly changed after the calcination procedure, while HRTEM images illustrated that there were amorphous layers with a thickness of ≈1 nm on the edge of WO_3_ nanosheets via calcination in both vacuum and H_2_ atmospheres (Figure 29b–d). The slight shift of (020) diffraction peak to a higher 2*θ* angle and the localized surface plasmon resonance (LSPR) peaks in the infrared region of the DRS spectra also proved the successful creation of OVs on the outside surface of WO_3_ nanosheets (Figure 29e,f). The free carrier density of WO_3_ nanosheets calcined in vacuum and H_2_ atmospheres was calculated to be 2.5 × 10^21^ and 2.0 × 10^21^ by the following Drude formula, respectively

(14) $$\begin{array}{c} \omega_{p}=\sqrt{\frac{N_{e}e^{2}}{\varepsilon_{0}m_{e}}} \end{array}$$

where *ω*_p_, *N*_e_, *e*, *ε*_0_, and *m*_e_ denote the bulk plasma frequency, charge carrier density, elementary charge, permittivity of free space, and effective mass of an electron, respectively. Under simulated solar light, WO_3_ nanosheets calcinated in H_2_ atmosphere demonstrated the highest O_2_ evolution rate of 1593 µmol h^−1^ g^−1^, ≈2.6‐fold higher than that of the pristine WO_3_ nanosheets (606 µmol h^−1^ g^−1^), which was attributed to the LSPR effect induced by the OVs that promoted the utilization of the solar energy (Figure 29g).^[^ ^188^ ^]^

### 3.4 Other Strategies

**Figure 30:**
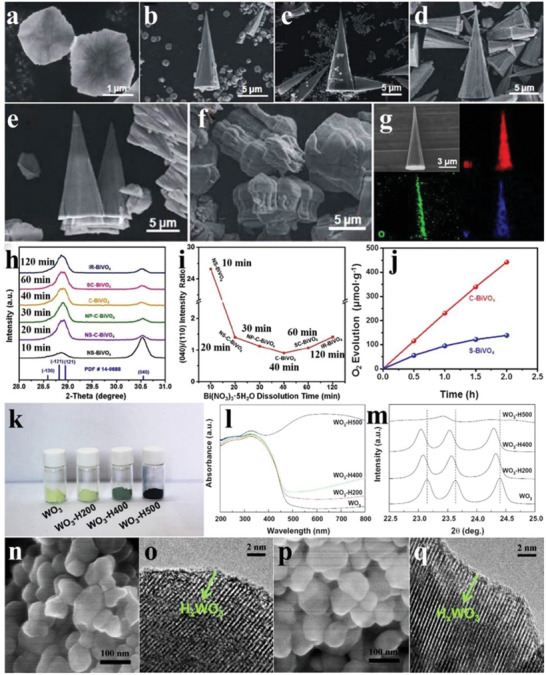
SEM images of BiVO_4_ with reaction time of a) 10 min, b) 20 min, c) 30 min, d) 40 min, e) 60 min, and f) 120 min. g) EDS mapping images of BiVO_4_ with reaction time of 40 min. h) Enlarged view of XRD patterns of BiVO_4_ with varieties of reaction times. i) Intensity ratios of (040)/(110) plane as a function of dissolution time of Bi(NO_3_)_3_·5H_2_O. j) Time courses of oxidation evolution for conical BiVO_4_ and BiVO_4_ nanosheets. Reproduced with permission.^[^ ^192^ ^]^ Copyright 2020, Royal Society of Chemistry. k) Digital picture, l) DRS, and m) local XRD patterns of pristine WO_3_ and WO_3_ with thermal treatment in varieties of temperatures under the H_2_ atmosphere. SEM images and HRTEM images of n,o) pristine WO_3_ and p,q) WO_3_ with thermal treatment in 200°C under the H_2_ atmosphere. Reproduced with permission.^[^ ^193^ ^]^ Copyright 2013, Elsevier.

Apart from the above strategies for improving the O_2_ evolution performances of photocatalysts, others methods including the formation of special microstructure, surface modification, and solid solution construction, can also effectively promote the O_2_ evolution production. Fabrication of photocatalysts with special morphology often brings advantages, such as more favorable visible light absorption, faster migration of photogenerated charge carriers and larger specific surface area.^[^ ^189^, ^190^, ^191^ ^]^ Since BiVO_4_ has the good morphological plasticity, BiVO_4_ with the unique conical shape was obtained by a simple two‐phase approach. The morphology of BiVO_4_ underwent a series of transformations during the synthetic process (**Figure** **30**a–g), and the most regular cone structure was observed after 40 min reaction. Besides, the dominantly exposed facets of BiVO_4_ changed as the reaction time increased (Figure 30h,i). Under visible light irradiation, the O_2_ evolution rate of as‐synthesized conical BiVO_4_ was three times that of BiVO_4_ nanosheets, which reflected the influence of formation of special microstructure on the photocatalytic performance of BiVO_4_ (Figure 30j).^[^ ^192^ ^]^

Li's group has investigated the influence of the hydrogenation treatment with a gradient of temperature for the O_2_ evolution rate of WO_3_ nanoparticles. The distinct thermal treatment temperatures led to the different colors of the samples (Figure 30k). Compared to pristine WO_3_, the hydrogenated WO_3_ showed stronger absorbance in visible light region (Figure 30l) and the obvious shift of the (002), (020), and (200) diffraction peaks to a lower 2*θ* angle due to the H_2_ intercalation effect within the crystal of WO_3_ (Figure 30m), whereas there was little impact on the morphology of WO_3_ (Figure 30n,p). The WO_3_ treated by H_2_ at 200°C exhibited the highest O_2_ evolution rate (75.3 µmol h^−1^), ≈2.3‐fold higher than the pristine WO_3_ (32.6 µmol h^−1^), benefiting from the formation of the H*_x_*WO_3_ layer around the edge of WO_3_ (Figure 30o,q).^[^ ^193^ ^]^

**Figure 31:**
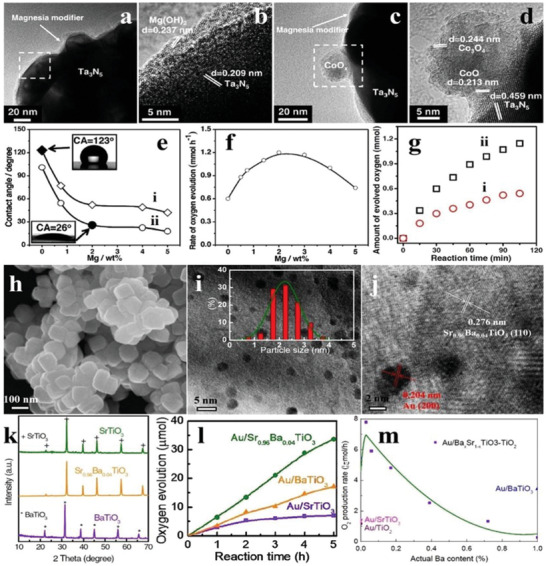
a,c) TEM and b,d) HRTEM images of a,b) Ta_3_N_5_–MgO and c,d) Ta_3_N_5_–MgO loaded with CoO*_x_*. e) Contact angles tests of i) Ta_3_N_5_–MgO and ii) Ta_3_N_5_–MgO loaded with CoO*_x_* as a function of the contents of magnesium. f)The O_2_ evolution rates of Ta_3_N_5_–MgO loaded with CoO*_x_* as a function of the contents of magnesium. g) Time courses of O_2_ evolution production for i) Ta_3_N_5_–MgO loaded with CoO*_x_* and ii) Ta_3_N_5_ loaded with CoO*_x_*. Reproduced with permission.^[^ ^194^ ^]^ Copyright 2015, Wiley. h) SEM image of Sr_0.96_Ba_0.04_TiO_3_ nanoparticles. i,j) TEM image, size distributions of Au (inset), and HRTEM image of Sr_0.96_Ba_0.04_TiO_3_ deposited with Au. k) XRD patterns of SrTiO_3_, BaTiO_3_, and Sr_0.96_Ba_0.04_TiO_3_. l) Time courses of O_2_ evolution production for Au/SrTiO_3_, Au/BaTiO_3_, and Au/Sr_0.96_Ba_0.04_TiO_3_. m) The O_2_ evolution rates of Sr_0.96_Ba_0.04_TiO_3_ as a function of doping amounts of barium. Reproduced with permission.^[^ ^196^ ^]^ Copyright 2017, Elsevier.

The hydrophilicity or hydrophobicity of the surface of photocatalysts also plays a key role in the interfacial contact between the photocatalyst and the loaded cocatalyst. In this regard, Li's group engineered the surface of Ta_3_N_5_ with MgO nanolayer as the modifier. TEM images showed that there was a layer of MgO deposited on the edge of Ta_3_N_5_ by calcination and subsequent nitridation treatment (**Figure** **31**a,b); it then quickly changed into Mg(OH)_2_ after contacting with the water molecules outside. The lattice spacing of 0.213 and 0.244 nm indicated the existence of both CoO and Co_3_O_4_ nanoparticles as the cocatalyst (denoted as CoO*_x_*) that were closely loaded on the surface of Ta_3_N_5_ (Figure 31c,d). When the amount of MgO was 2%, the contact angle between Ta_3_N_5_ and CoO*_x_* reached the saturated value and the catalyst exhibited the highest O_2_ evolution rate of 1.2 mmol h^−1^ (Figure 31e,f). Ta_3_N_5_ modified by MgO displayed a better water oxidation performance than pristine Ta_3_N_5_ (Figure 31g), which was attributed to the formation of the MgO layer on the surface of Ta_3_N_5_ that improved the deposition of CoO*_x_*, leading to the enhancement of the separation efficiency of photogenerated charge carriers.^[^ ^194^ ^]^

Besides, construction of solid solutions was considered to be an effective way for controllable adjustment of bandgap and energy levels.^[^ ^195^ ^]^ Ye's group incorporated a certain proportion of barium into SrTiO_3_ by the hydrothermal approach to construct the Sr_0.96_Ba_0.04_TiO_3_ solid solution. The diameter of the as‐synthesized solid solution nanoparticles was ≈100 nm (Figure 31h) with the crystal structure kept the same to the original SrTiO_3_ and BaTiO_3_ (Figure 31k). The size distributions of Au nanoparticles as cocatalyst deposited on the surface of the solid solution was between 1 and 4 nm (Figure 31i,j). Under visible light irradiation, Sr_0.96_Ba_0.04_TiO_3_ showed the highest O_2_ evolution rate (6.7 µmol h^−1^) when 3% Ba was doped, ≈4.79 and 1.97‐fold higher than that of pure SrTiO_3_ and BaTiO_3_, respectively. The enhanced O_2_ production activity was mainly attributed to the optimized energy level of SrTiO_3_ by the Ba doping (Figure 31l,m).^[^ ^196^ ^]^ The O_2_ evolution production performance of strategic photocatalysts under distinct reaction conditions is shown in **Table** **2**.

**Table 2:** Strategies for enhancing photocatalytic O_2_ evolution of semiconductor photocatalysts

Photocatalyst	Sacrificial agent	Amount of sacrificial agent	Light source	Incident light [nm]	Oxygen production rate	AQE	Refs.
CoO*_x_*–TiO_2_	NaIO_3_	20 × 10^−3^ m	150 W UV	–	47 µmol h^−1^	15.5% at 365 nm	^[^ ^140^ ^]^
CoO*_x_*–TiO_2_	AgNO_3_	100 × 10^−3^ m	UV light	–	34.42 µmol h^−1^	–	^[^ ^197^ ^]^
Ir–TiO_2_	KIO_3_	1.5 × 10^−3^ m	400 W UV	–	≈140 µmol g^−1^	–	^[^ ^198^ ^]^
Carbon dots/TiO_2_	AgNO_3_	15 mg	114 W UV	–	3598 µmol h^−1^ g^−1^	–	^[^ ^199^ ^]^
TiO_2_@CoAl‐LDH	AgNO_3_	10 mg	300 W Xe	–	2.34 mmol h^−1^ g^−1^	–	^[^ ^153^ ^]^
FeTiO_3_–TiO_2_	AgNO_3_	10 × 10^−3^ m	300 W Xe	–	71 µmol h^−1^	9.1% at 420 nm	^[^ ^154^ ^]^
TiO_2_/SrTiO_3_	AgNO_3_	–	300 W Xe	–	103 µmol h^−1^	35.4% at 365 nm	^[^ ^200^ ^]^
g‐C_3_N_4_/a‐TiO_2_/r‐TiO_2_	AgNO_3_	10 × 10^−3^ m	Sunlight	–	198.8 µmol h^−1^	–	^[^ ^201^ ^]^
WO_3_–TiO_2_/Nb_2_O_5_	Fe_2_(SO_4_)_3_	–	250 W Hg	–	151.8 µmol L^−1^ h^−1^	–	^[^ ^202^ ^]^
Ag_3_PO_4_/CeO_2_/TiO_2_	–	–	300 W Xe	≤400	23.13 µmol h^−1^	–	^[^ ^203^ ^]^
B^3+^–TiO_2_	AgNO_3_	16.7 × 10^−3^ m	–	–	≈50 µmol h^−1^	–	^[^ ^172^ ^]^
Ta^5+^–TiO_2_	AgNO_3_	0.45 × 10^−3^ m	UV light	–	≈21 µmol h^−1^	–	^[^ ^173^ ^]^
Ta/N–TiO_2_	NaIO_3_	100 × 10^−6^ m	360 W Xe	≥420	≈15 µmol h^−1^	0.5% at 420 nm	^[^ ^179^ ^]^
Ta/N–TiO_2_	FeCl_3_	1 × 10^−3^ m	300 W Xe	≥400	≈13 µmol h^−1^	–	^[^ ^180^ ^]^
S–TiO_2_	Fe(OH)_3_	–	500 W Xe	≥350	≈2.9 µmol h^−1^	–	^[^ ^204^ ^]^
Ta/N–TiO_2_	AgNO_3_	10 × 10^−3^ m	300 W Xe	≥400	≈5.4 µmol h^−1^	–	^[^ ^205^ ^]^
Pt–Co_3_O_4_/BiVO_4_	NaIO_3_	20 × 10^−3^ m	300 W Xe	≥420	≈160 µmol h^−1^	–	^[^ ^142^ ^]^
Co_4_O_4_(O_2_CMe)_4_CNpy_4_–BiVO_4_	NaIO_3_	10 × 10^−3^ m	300 W Xe	≥420	≈51 µmol h^−1^	4.5% at 420 nm	^[^ ^144^ ^]^
Pt/MnO*_x_*/BiVO_4_	NaIO_3_	20 × 10^−3^ m	300 W Xe	≥420	≈660 µmol h^−1^ g^−1^	–	^[^ ^61^ ^]^
FeOOH/BiVO_4_	AgNO_3_	850 mg	300 W Xe	≥400	67.2 µmol h^−1^	2.4% at 420 nm	^[^ ^143^ ^]^
Au/CoO*_x_*/BiVO_4_	K_3_[Fe(CN)_6_]	10 × 10^−3^ m	300 W Xe	≥420	32 µmol h^−1^	10.3% at 420 nm	^[^ ^206^ ^]^
BiVO_4_/MCM‐41	AgNO_3_	–	300 W Xe	–	172.8 µmol h^−1^	–	^[^ ^207^ ^]^
BiVO_4_/B–g‐C_3_N_4_	AgNO_3_	5 × 10^−3^ m	150 W Xe	≥420	1027.2 µmol h^−1^ g^−1^	–	^[^ ^156^ ^]^
BiVO_4_/ZnO	AgNO_3_	50 × 10^−3^ m	300 W Xe	≥420	68 µmol h^−1^	5.0% at 450 nm	^[^ ^160^ ^]^
BiVO_4_@Cu_2_O	AgNO_3_	50 × 10^−3^ m	300 W Xe	≥420	1500 µmol h^−1^ g^−1^	–	^[^ ^208^ ^]^
BiVO_4_/g‐C_3_N_4_	AgNO_3_	50 × 10^−3^ m	300 W Xe	–	600 µmol h^−1^ g^−1^	2.36%	^[^ ^161^ ^]^
CeVO_4_/rGO/BiVO_4_	AgNO_3_	1000 mg	–	–	85.68 µmol L^−1^ g^−1^	–	^[^ ^209^ ^]^
C–BiVO_4_	AgNO_3_	4.5 × 10^−3^ m	300 W Xe	≥420	800 µmol L^−1^	–	^[^ ^174^ ^]^
Mo–BiVO_4_	AgNO_3_	50 × 10^−3^ m	Sunlight	–	942 µmol h^−1^ g^−1^	–	^[^ ^210^ ^]^
Conical BiVO_4_	AgNO_3_	25 × 10^−3^ m	300 W Xe	≥400	230 µmol h^−1^ g^−1^	–	^[^ ^192^ ^]^
Pt/RuO_2_/WO_3_	NaIO_3_	10 × 10^−3^ m	300 W Xe	≥420	41 µmol h^−1^	14.4% at 420 nm	^[^ ^131^ ^]^
B_2_O_3−_ *_x_*N*_x_*@WO_3_	AgNO_3_	850 mg	–	≥400	74.5 µmol h^−1^	–	^[^ ^146^ ^]^
WO_3_/graphene	Fe_2_(SO_4_)_3_	10 × 10^−3^ m	150 W Xe	≥400	40 µmol L^−1^ h^−1^	–	^[^ ^147^ ^]^
WO_3_@graphene	Fe_2_(SO_4_)_3_	0.16 × 10^−3^ m	300 W Xe	–	43.1 µmol L^−1^ h^−1^	–	^[^ ^148^ ^]^
Ru–WO_3_	NaIO_3_	5 × 10^−3^ m	300 W Xe	≥300	65 µmol h^−1^	–	^[^ ^211^ ^]^
WO_3_/rGO	AgNO_3_	30 × 10^−3^ m	300 W Xe	–	580 µmol h^−1^ g^−1^	–	^[^ ^212^ ^]^
WO_3_/BiVO_4_	–	–	300 W Xe	≥420	20 µmol h^−1^ mg^−1^	–	^[^ ^155^ ^]^
WO_3_/TiO_2_	–	–	300 W Xe	–	178 µmol h^−1^ g^−1^	1.8% at 400 nm	^[^ ^213^ ^]^
WO_3_/Ag_3_PO_4_	AgNO_3_	510 mg	300 W Xe	≥420	≈425 µmol h^−1^ g^−1^	–	^[^ ^214^ ^]^
WO_2_–WO_3_	AgNO_3_	10 × 10^−3^ m	500 W Xe	≥400	265 mmol h^−1^ g^−1^	–	^[^ ^215^ ^]^
Cs–WO_3_	AgNO_3_	–	300 W Xe	≥420	51.3 µmol h^−1^	3.7%	^[^ ^175^ ^]^
WO_3−_ *_x_*–V/HT	NaIO_3_	10 × 10^−3^ m	Sunlight	–	1593 µmol h^−1^ g^−1^	9.3% at 420 nm	^[^ ^188^ ^]^
S–WO_3_	–	–	250 W Xe	≥500	76.7 µmol L^−1^ h^−1^ g^−1^	–	^[^ ^216^ ^]^
H*_x_*WO_3_–WO_3_	AgNO_3_	10 × 10^−3^ m	300 W Xe	≥420	75.3 µmol h^−1^	–	^[^ ^193^ ^]^
Au–*α*‐Fe_2_O_3_	Na_2_S_2_O_8_	5.33 × 10^−3^ m	300 W Xe	≥420	10.81 µmol h^−1^	–	^[^ ^132^ ^]^
*α*‐Fe_2_O_3_/rGO	AgNO_3_	68 mg	300 W Xe	–	752 µmol h^−1^ g^−1^	–	^[^ ^149^ ^]^
Fe_2_O_3_/C–C_3_N_4_	AgNO_3_	10 × 10^−3^ m	–	≥420	22.3 µmol h^−1^	–	^[^ ^162^ ^]^
Pt–Bi_4_NbO_8_Cl	FeCl_3_	2 × 10^−3^ m	300 W Xe	≥400	≈96 µmol h^−1^	2.5% at 420 nm	^[^ ^100^ ^]^
MoO_3_–Bi_4_TaO_8_Cl	AgNO_3_	5 × 10^−3^ m	300 W Xe	≥420	≈28 µmol h^−1^	25% at 420 nm	^[^ ^103^ ^]^
Fe/Ru–Bi_4_TaO_8_Cl	FeCl_3_	4 × 10^−3^ m	300 W Xe	≥400	26 µmol h^−1^	1.6% at 420 nm	^[^ ^217^ ^]^
Ru–TaON	NaIO_3_	1 × 10^−3^ m	300 W Xe	≥420	13.0 µmol h^−1^	–	^[^ ^135^ ^]^
Rh/Ru–TaON	NaIO_3_	1 × 10^−3^ m	300 W Xe	≥400	≈46.67 µmol h^−1^	6.9% at 420 nm	^[^ ^136^ ^]^
Co_3_O_4_@SBA^a)^	Na_2_SO_4_/Na_2_S_2_O_8_	975 mg/357 mg	300 W Xe	≥450	≈135 µmol h^−1^	–	^[^ ^137^ ^]^
CoPOM@MIL‐101^b)^	Na_2_S_2_O_8_	0.375 × 10^−3^ m	300 W Xe	≥420	≈68.38 µmol h^−1^	–	^[^ ^145^ ^]^
Ru/Co–TaON	NaIO_3_	1 × 10^−3^ m	300 W Xe	≥400	≈12.5 µmol h^−1^	–	^[^ ^218^ ^]^
Ir/TiO_2_/Ta_3_N_5_	NaIO_3_	1 × 10^−3^ m	300 W Xe	≥420	24.1 µmol h^−1^	–	^[^ ^219^ ^]^
Rh–KCa_2_Nb_3_O_10_	NaIO_3_	5 × 10^−3^ m	300 W Xe	≥300	≈3.4 µmol h^−1^	–	^[^ ^107^ ^]^
Bi_2_MoO_6_/Ru/g‐C_3_N_4_	NaIO_3_	396 mg	300 W Xe	≥420	328.34 µmol h^−1^ g^−1^	–	^[^ ^167^ ^]^
N–KCa_2_Nb_3_O_10_	AgNO_3_	10 × 10^−3^ m	300 W Xe	≥400	≈1.92 µmol h^−1^	–	^[^ ^220^ ^]^
CoO*_x_*/MgO–Ta_3_N_5_	AgNO_3_	1500 mg	300 W Xe	≥420	1.2 mmol h^−1^	11.3% at 500 nm	^[^ ^194^ ^]^
Au/Sr_0.96_Ba_0.04_TiO_3_	AgNO_3_	5 × 10^−3^ m	300 W Xe	≥400	6.7 µmol h^−1^	–	^[^ ^196^ ^]^
Fe‐TAML^c)^	Na_2_S_2_O_8_	8.3 × 10^−3^ m	Sunlight	–	0.79 µmol h^−1^	–	^[^ ^221^ ^]^

^a)^ SBA = Mesoporous silica

^b)^ COPOM = [Co_4_(H_2_O)_2_(PW_9_O_34_)_2_]^10−^

^c)^ Fe‐TAML = Biuret modified tetra‐amidomacrocyclic ligands.

## 4 Conclusions, Challenges, and Perspectives

Under the social background of advancing the sustainable development of energy resources, the photocatalytic water splitting has been gradually become the focus due to the features of abundant resources, environmental‐friendly, etc. As the rate‐determining step of the water splitting reaction, water oxidation is the key bottleneck that restricts the efficiency during this process. This review summarizes the latest research progresses of photocatalytic water oxidation. The content includes the introduction of several classical water oxidation photocatalysts (e.g., TiO_2_, BiVO_4_, WO_3_), featuring the crystalline structures, synthesis approaches, and morphologies. On this basis of the critical issues that hinder the photocatalytic activity of photocatalysts, such as the low utilization of visible light and fast recombination of photogenerated charge carriers, the corresponding effective solutions, including the cocatalyst loading, heterojunction construction, doping and vacancy formation, and other strategies, are summarized.

In the last ten years, although a series of oxygen evolution photocatalysts have been developed, there is still a long way to go before the practical industrial applications. The photocatalysts for oxygen evolution still suffer from low efficiency or poor physicochemical stability, and especially most of them require the presence of sacrificial agents and cocatalysts, which also undoubtedly increase the economic costs to the industrial applications. In the future, more efforts are in need:

i) The development of efficient water oxidation photocatalysts is still the present research focus, and the Sillén–Aurivillius perovskites will show significant potential for photocatalytic O_2_ evolution. First, the VB of these perovskites such as Bi_4_NbO_8_Cl is mainly occupied by the O 2p orbitals. It makes them not be easily corroded by the photogenerated holes, thus demonstrating high photochemical stability. Second, the synthesis methods of this kind of materials are diverse, including 1SSR, 2SSR, 2PC, and flux, which allows the preparation process to be flexible and easy to optimize the photocatalytic performance. Additionally, the Sillén–Aurivillius perovskites have diverse compositions, which enables the adjustable light absorption or bandgap and photocatalytic O_2_ evolution activity. Therefore, the rational design strategies based on crystal structure and band structure are expected to yield high‐performance perovskites for O_2_ evolution in the future.
ii) At present, the strategies for O_2_ evolution performance enhancement are mainly achieved by improving the light absorption ability and charge separation efficiency, whereas the researches on the surface catalytic reaction are rarely involved. Actually, the reactive sites of photocatalysts are closely related to the adsorption of reactants and the reaction activation energy. For example, the construction of the surface defects can obviously enrich the active sites of photocatalysts. Therefore, the exploration of the catalytic active sites for O_2_ evolution is expected to be one of the research priorities in the future.
iii) Most of the reported works mainly focused on promoting the separation efficiency of photogenerated charge carriers to achieve the purpose of improving the O_2_ evolution performance. However, less attention has been paid to the physicochemical stability of photocatalysts during the photocatalytic reaction process, whereas the actual situation is that the physicochemical stability of most of as‐synthesized materials is often affected by many factors, such as the synthetic routes and conditions. In addition, some O_2_ evolution photocatalysts are prone to be self‐poisoned by the photogenerated holes, which results in the inactivation after photoreactions. Thus, developing effective tactics for improving the chemical and physicochemical stability of O_2_ evolution photocatalysts are necessary.

Besides, the spatial separation for the occurrence of reduction and oxidation reactions should be considered, which can effectively inhibit the inverse reaction that usually occurs on the surface of photocatalyst. For instance, the hydrogen farm strategy proposed by Li's group is a promising direction,^[^ ^222^ ^]^ and in which we believe more breakthroughs will be achieved.

## Conflict of Interest

The authors declare no conflict of interest.
